# Comparative transcriptome profiling provides insights into plant salt tolerance in seashore paspalum (*Paspalum vaginatum*)

**DOI:** 10.1186/s12864-020-6508-1

**Published:** 2020-02-07

**Authors:** Peipei Wu, Steven Cogill, Yijian Qiu, Zhigang Li, Man Zhou, Qian Hu, Zhihui Chang, Rooksana E. Noorai, Xiaoxia Xia, Christopher Saski, Paul Raymer, Hong Luo

**Affiliations:** 1 Department of Genetics and Biochemistry, Clemson University, Clemson, SC 29634 USA; 2 Present address: Emergency Medicine, Stanford University, Stanford, California 94305 USA; 3 Clemson University Genomics Institute, Clemson University, Clemson, SC 29634 USA; 4 Department of Crop & Soil Sciences, University of Georgia, Griffin, GA 30223 USA

**Keywords:** Ca^2+^-signaling, *Paspalum vaginatum*, Potassium retention, RNA-seq, Salt stress, Transcriptome profiling, Vacuolar sequestration

## Abstract

**Background:**

Seashore paspalum (*Paspalum vaginatum*), a halophytic warm-seasoned perennial grass, is tolerant of many environmental stresses, especially salt stress. To investigate molecular mechanisms underlying salinity tolerance in seashore paspalum, physiological characteristics and global transcription profiles of highly (Supreme) and moderately (Parish) salinity-tolerant cultivars under normal and salt stressed conditions were analyzed.

**Results:**

Physiological characterization comparing highly (Supreme) and moderately (Parish) salinity-tolerant cultivars revealed that Supreme’s higher salinity tolerance is associated with higher Na^+^ and Ca^2+^ accumulation under normal conditions and further increase of Na^+^ under salt-treated conditions (400 mM NaCl), possibly by vacuolar sequestration. Moreover, K^+^ retention under salt treatment occurs in both cultivars, suggesting that it may be a conserved mechanism for prevention of Na^+^ toxicity. We sequenced the transcriptome of the two cultivars under both normal and salt-treated conditions (400 mM NaCl) using RNA-seq. De novo assembly of about 153 million high-quality reads and identification of Open Reading Frames (ORFs) uncovered a total of 82,608 non-redundant unigenes, of which 3250 genes were identified as transcription factors (TFs). Gene Ontology (GO) annotation revealed the presence of genes involved in diverse cellular processes in seashore paspalum’s transcriptome. Differential expression analysis identified a total of 828 and 2222 genes that are responsive to high salinity for Supreme and Parish, respectively. “Oxidation-reduction process” and “nucleic acid binding” are significantly enriched GOs among differentially expressed genes in both cultivars under salt treatment. Interestingly, compared to Parish, a number of salt stress induced transcription factors are enriched and show higher abundance in Supreme under normal conditions, possibly due to enhanced Ca^2+^ signaling transduction out of Na^+^ accumulation, which may be another contributor to Supreme’s higher salinity tolerance.

**Conclusion:**

Physiological and transcriptome analyses of seashore paspalum reveal major molecular underpinnings contributing to plant response to salt stress in this halophytic warm-seasoned perennial grass. The data obtained provide valuable molecular resources for functional studies and developing strategies to engineer plant salinity tolerance.

## Background

High salinity stress, which is one of the most severe environmental stresses, impairs crop production on at least 20% of the cultivated land worldwide [1]. This problem becomes increasingly severe due to the rising sea level from global warming and inappropriate irrigation practice. Salinity inflicts not only ionic stress but also osmotic stress on plants. As a consequence of these primary effects, secondary stresses such as oxidative stress often occur [2]. To survive against these stresses, plants have evolved a complex of mechanisms involving multiple genes and strategies at physiological, molecular and metabolic levels [3]. As high levels of cytosolic Na^+^ are toxic to plants by interfering with cellular K^+^/Na^+^ homeostasis and inhibiting enzyme activities, plants utilize three major mechanisms to prevent excess Na^+^ accumulation in the cytoplasm: restriction of Na^+^ entry into the cells, exclusion of Na^+^ out of the cells and compartmentalization of excessive Na^+^ into the vacuoles. Two types of plasma membrane localized High-affinity K^+^ Transporter (HKT) are important salt tolerance determinants by regulating transportation of Na^+^ and K^+^. The Class 1 HKT transporters mediate Na^+^-selective transport. The current model in *Arabidopsis* suggests that the Class 1 HKT transporter AtHKT1 plays an essential role in protecting leaf blades from excessive accumulation of Na^+^ by unloading of Na^+^ from the xylem sap [4]. The Class 2 HKT transporters are suggested to mediate both Na^+^ and K^+^ transport [5]. Study of a Class 2 HKT transporter OsHKT2;1 in rice demonstrated a fail-safe mechanism of Na^+^ uptake under K^+^ starved rice roots [6]. The plasma membrane localized Na^+^/H^+^ transporter Salt Overly Sensitive 1 (SOS1) and the tonoplast localized Na^+^/H^+^ transporter NHX are another two important determinants for maintaining low cytosolic Na^+^ concentration in plant cells by exporting Na^+^ out of the cell and sequestration of Na^+^ into the vacuoles, respectively [7, 8].

To neutralize the negative effect of osmotic stress imposed by high concentration of salt, plants can accumulate compatible solutes (e.g. proline, glycine betaine, sugars, mannitol, myo-inositol) and proteins (e.g. Late-embryogenesis-abundant-proteins (LEAs) and dehydrins) for osmotic adjustment or other protective functions [9]. Most of the abiotic stress types including salinity disrupt the balance of cellular metabolism, resulting in oxidative stress with elevated level of reactive oxygen species (ROS), such as the superoxide radical anion (O_2_˙^−^), hydrogen peroxide (H_2_O_2_), and hydroxyl radicals (OH˙). The elevated level of ROS plays a dual role in the salinity responses of plants. On one hand, the enhanced production of ROS is toxic to plants as they can cause protein and membrane lipid peroxidation, and DNA and RNA damage [10]. To ensure survival, plants have developed two efficient antioxidant defense systems to work in concert for ROS scavenging, which include both enzymatic and non-enzymatic machinery. Major enzymatic components include catalase (CAT), superoxide dismutase (SOD), ascorbate peroxidase (APX), glutathione peroxidase (GPX) and dehydroascorbate reductase (DHAR) while non-enzymatic antioxidants include ascorbic acid (AA), glutathione (GSH), phenolic compounds [11, 12]. On the other hand, ROS can also act as a pivotal signaling molecule to trigger tolerance against stress [13]. For example, loss-of-function of one of the NADPH oxidase members AtrbohF, which catalyzes the production of ROS in root vasculature systems, leads to salt hypersensitivity phenotype due to the elevated root-to-shoot delivery of soil Na^+^ and consequently elevated shoot Na^+^ levels [14].

The plant kingdom has about 1% of plant species classified as halophytes that possess capacities for salt tolerance of around 200 mM NaCl or more as a result of evolutionary adaptation to their habitats [15]. The inherent potentiality of halophytes to counteract the negative impact of salinity stress makes it very interesting and promising to investigate the associated mechanisms. Seashore paspalum (*Paspalum vaginatum*) is a halophytic warm-season perennial grass of the *Poaceae* family, which is native to tropical and coastal regions worldwide and is among the most salinity-tolerant turfgrass species [16, 17]. Previous studies show that its superior salinity tolerance is attributed to the maintenance of photosynthesis, shoot growth rate and tissue water content through osmotic adjustment [16, 17]. However, little is known about the molecular mechanisms underlying its high salinity tolerance and the limited genomic information of seashore paspalum has impeded further investigation. A recent study using the combination of 2-DE and MS technologies linked ROS detoxification and ATP biosynthesis to the superior salinity tolerance in seashore paspalum’s roots [18]. Another recent study using RNA-seq provided the global transcriptome data for the seashore paspalum cultivar ‘Adalady’ for the first time [19]. However, no study has reported how the different cultivars of seashore paspalum with inherent variation in their capabilities of salt tolerance undergo dynamic change of ion accumulation and how they respond to salt stress globally at the transcriptome level. This will help us better understand plant salinity tolerance mechanism at the physiological and molecular level and identify salt stress-related genes for functional study and application in the future.

In this study, we monitored the dynamic change of Na^+^, K^+^ and Ca^2+^ accumulation before and after salt treatment comparing two cultivars of seashore paspalum. One is called Supreme, which is the most salinity-tolerant cultivar of all commercially grown paspalums (http://georgiacultivars.com/cultivars/seaisle-supreme-paspalum). Another cultivar is called Parish, which is a moderately salinity-tolerant cultivar. We also applied RNA-seq analysis to reveal differences in gene expression between two cultivars under normal conditions and when they are exposed to salt stress. To our knowledge, this study provides the first transcriptome profile for seashore paspalum under salt stress. By comparing ion dynamics and expression profiling data of the two cultivars under both non-stressed and salt-stressed conditions, this study provides a new insight into the physiological and molecular mechanisms of high salinity tolerance in halophytes and establishes a solid foundation for future studies of genes involved in salinity tolerance.

## Results

### Ion dynamics of supreme and parish under normal and salt-treated conditions

**Fig. 1:**
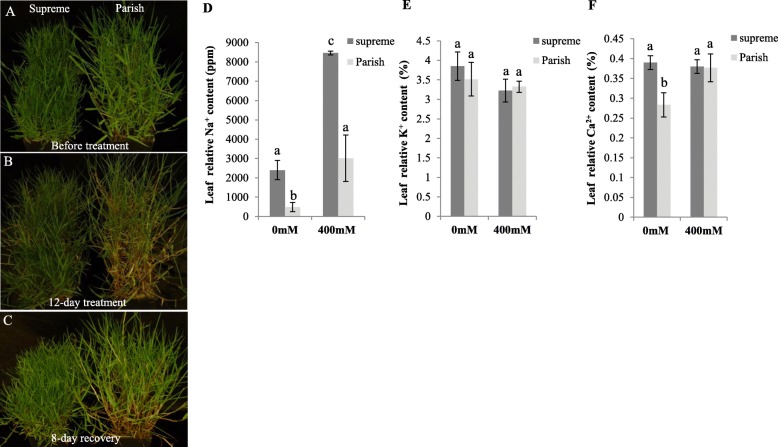
Responses of Supreme and Parish to salt treatment. **a** 8-week Supreme and Parish grown from the same number of tillers before salt treatment. **b** Performance of Supreme and Parish at a 12-day treatment of 400 mM NaCl. **c** Performance of Supreme and Parish 8 days after recovery from a 12-day treatment of 400 mM NaCl. **d** Leaf Na^+^ content under normal conditions and 400 mM NaCl treatment. **e** Leaf K^+^ content under normal conditions and 400 mM NaCl treatment. **f** Leaf Ca^2+^content under normal conditions and 400 mM NaCl treatment. The statistically significant difference was determined by one-way ANOVA analysis. Groups not sharing the same letter show statistically significant difference (*P* < 0.05)

Many studies have shown that seashore paspalum is among the most salinity-tolerant warm-season turfgrass species with a NaCl tolerance threshold of 474.0 mM [20]. To study the mechanisms underlying seashore paspalum’s high salt tolerance, two cultivars, Supreme and Parish were used for morphological, physiological and comparative transcriptomics studies (Fig. 1a). Firstly, we compared their morphological differences in response to salt treatment. Supreme and Parish grown under the same conditions were exposed to 400 mM NaCl solution. After a 12-day treatment, chlorotic leaves were clearly observed in Parish while Supreme was not strongly affected, indicative of a more tolerant trait of Supreme than Parish (Fig. 1b). Moreover, Supreme also has better recovery than Parish after salt treatment based on chlorosis in leaves (Fig. 1c). To reveal possible physiological mechanisms of differential performance of Supreme and Parish under salt stress, we measured their leaf ion contents under normal and salt-stressed conditions. Supreme has significantly higher Na^+^ content than Parish under both conditions, whereas their K^+^ contents are similar, and remain the same even upon exposure to salinity (Fig. 1d, e). In addition, Supreme has significantly higher Ca^2+^ content than Parish under normal conditions, but their Ca^2+^ contents are similar after treatment with salt (Fig. 1f). The demonstration of higher salt tolerance of Supreme and its physiological characteristics implies the importance of the associated genetic underpinnings.

### Transcriptome sequencing of supreme and parish under normal and salt-treated conditions

To characterize and compare the transcriptome response of Supreme and Parish under salt treatment, we treated plants with 400 mM NaCl for 1 h. We use this condition because it was suggested that genes that rapidly changed expression upon salt stress should be important for salt tolerance [21]. Illumina sequencing of indexed and pooled RNA with polyA tails generated a total of 80.29 million and 78.88 million paired-end reads with a single read length about 101 bp for Supreme and Parish, respectively. An overview of the sequencing and assembly results are represented in Additional file 1: Table S1. Among these raw reads, 95.89 and 95.77% remained after trimming for Supreme and Parish, respectively, which were then de novo assembled into one reference transcriptome using Trinity. De novo assembly of mixed trimmed reads generated 342,165 Trinity transcripts (the individual assembled contig) with an average length of 784 bp and N50 value of 1339 bp, and a total of 244,926 Trinity genes (the clustered Trinity transcripts based on shared sequence content) with average length of 580 bp and N50 value of 761 bp. GC content, which is an important indicator of the gene and genomic composition as well as DNA stability is 49.7% in seashore paspalum’s transcriptome, which is similar to the transcriptome GC composition of other monocot plants such as rice (51.1%) and *Triticum aestivum* (51.4%) [22, 23].

A total of 169,391 ORFs (49.5% of all Trinity transcripts) were identified among 342,165 Trinity transcript sequences using TransDecoder. Using CD-HIT software, the 169,391 ORFs were clustered into 82,608 unigenes. The length distribution of the unigenes is shown in Additional file 1: Figure S1. Approximately 48.4 and 20.5% of the unigenes had a length > = 500 bp and > = 1000 bp, respectively. To compare with the previously reported transcriptome with 32,603 reported Trinity genes assembled in another seashore paspalum cultivar “Adalady”, we conducted the Benchmarking Universal Single Copy Orthologs (BUSCO) analysis to check the quality and completeness of assembly. By searching 3278 total BUSCO groups against our transcriptome, 3, 028 (92.3%) were “complete”, 174 (5.3%) were “fragmented”, and the remaining 76 (2.4%) were “missing”, indicating the high completeness of our assembled transcripts. As shown in Additional file 1: Table S3, the transcriptome assembled in this study has a higher completeness and quality than the previously reported transcriptome, thus providing additional genomic resources that can be exploited for gene discovery and functional study [19].

### Functional annotation of seashore paspalum’s transcriptome

Homology-based functional annotation of the seashore paspalum unigenes was then carried out. Distribution of the annotated unigenes in each database is shown in Additional file 1: Table S2. 82,608 unigenes were blasted against the NCBI non-redundant (nr) protein database using Blastx. 65,540 (79.3%) out of the 82,608 unigenes showed homology to the nr protein sequences. E-value distribution of blast results is shown in Additional file 1: Figure S2. The best blastx hits against the nr database were then imported to Blast2GO software [24] for gene ontology (GO) classification and the result is shown in Additional file 1: Figure S3. Among 82,608 unigenes, 36,387 unigenes (44%) were successfully annotated with 16 GO terms (level 2) and classified into three ontologies: biological process (BP, Additional file 1: Figure S3A), cellular component (CC, Additional file 1: Figure S3B), and molecular function (MF, Additional file 1: Figure S3C). Within the BP category, genes involved in metabolic process (16946), cellular response (14342), single-organism process (8922) and biological regulation (3787) are highly represented. The CC category mainly comprises genes involved in membrane (10287), cell (10050), cell part (9904), membrane part (8528) and organelle (6716). Under MF, catalytic activity (15615) was the most abundant GO term, followed by binding (15411).

To compare the gene repertoire of seashore paspalum to other plant species, we aligned the unigenes against the nr protein database and performed the species distribution of the unigenes using Blast2GO software. As shown in Additional file 1: Figure S4, the five top-hit species that best match the sequences of seashore paspalum unigenes are *Setaria italica*, *Sorghum bicolor*, *Zea mays*, *Oryza sativa Japonica* Group and *Brachypodium distachyon*, all of which belong to the *Poaceae* family.

### Identification of transcription factors in seashore paspalum’s transcriptome

**Fig. 2:**
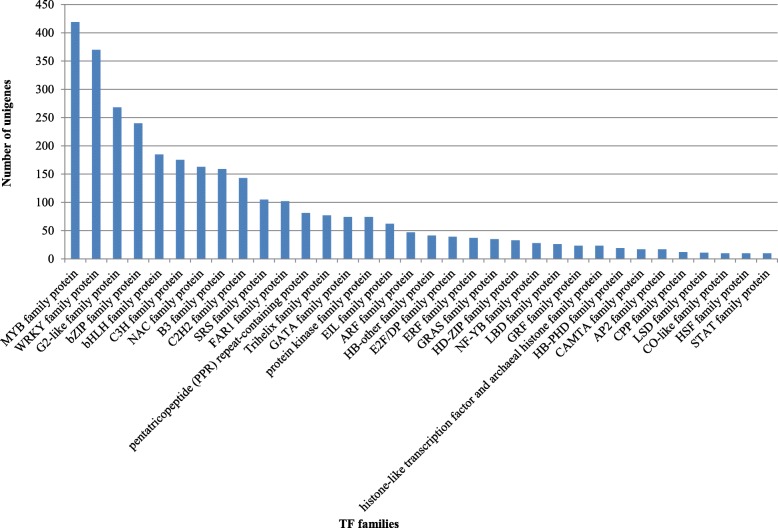
Distribution of transcription factors (TFs) in seashore paspalum’s transcriptome. A total of 3250 TF unigenes were identified by blastx against *Arabidopsis* and rice TF database with an E-value cutoff of 1E^−5^. Thirty-four TF families with ten or more unigenes were plotted

Transcription factors (TFs) play a vital role in regulating plant stress response as important regulatory elements. To identify potential TFs in the seashore paspalum’s transcriptome, 82,608 unigenes were searched against the PlantTFDB [25, 26] using Blastx. There are 3250 transcripts that have at least one hit to the *Arabidopsis* and *Oryza* TFs, representing about 4% of the total unigenes and covering 68 putative TF families (Additional file 1: Table S4). The TF gene families with ten or more unigenes identified in the seashore paspalum transcriptome are presented in Fig. 2, among which the five most abundant categories are Myb (419), followed by WRKY (370), G2-like (268), bZIP (240), and bHLH (185).

### Differentially expression analysis for supreme and parish under salt treatment

**Fig. 3:**
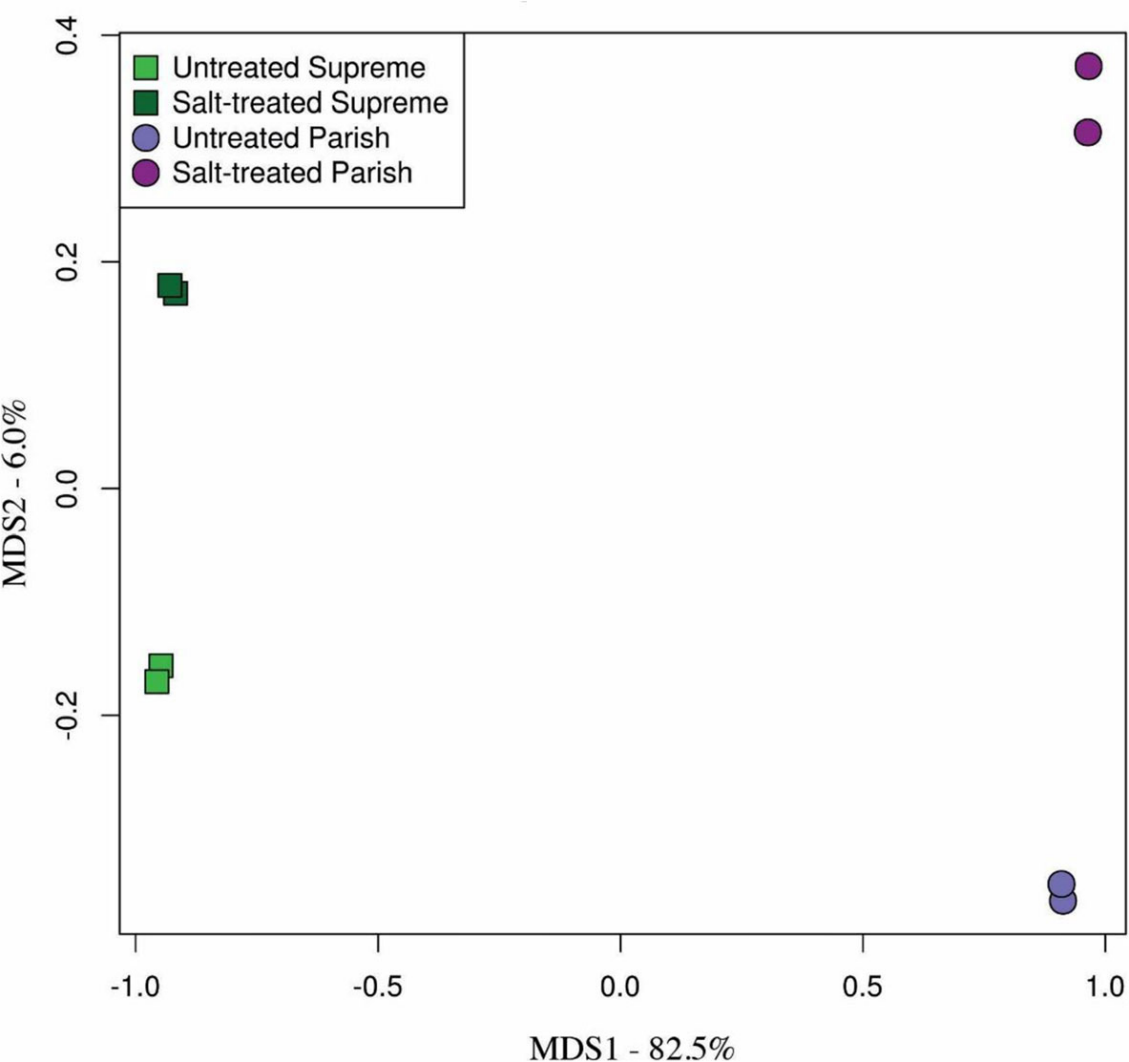
MDS plot showing reproducibility among two biological replicates of our RNA-seq samples. The MDS plot was generated by using the expected counts generated by RSEM to ordinate samples in multidimensional space based on differences in expression values. The close clustering of biological replicates indicates a high degree of consistency across all genes. The percentage of variance in the X axis indicating the difference of the two plant types is 82.5% while the percentage of variance in the Y axis indicating the difference of non-treated and salt-treated samples is 6.0%

**Fig. 4:**
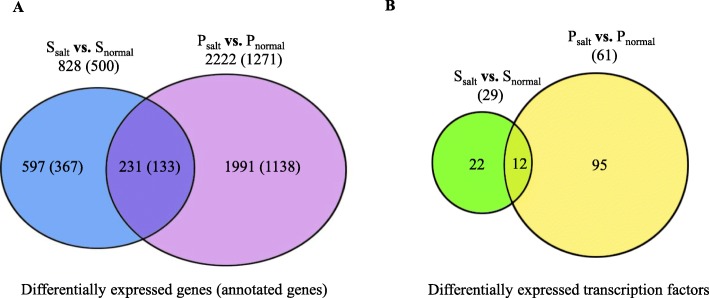
Venn diagram showing the number of common and specific DEGs with 2-fold change or above for Supreme and Parish under salt treatment. The number of common and specific DEGs (**a**) and transcription factors (**b**) with 2-fold change or above, and an adjusted P value ≤0.01 were shown in the overlapping and non-overlapping regions, respectively. Numbers within parentheses represent DEGs that have assigned GO terms. S_normal_: untreated Supreme; S_salt_: salt-treated Supreme; P_normal_: untreated Parish; P_salt_: salt-treated Parish

To compare gene expression levels in the control and salt-treated samples, the trimmed reads in each library were mapped to the 82,608 reference unigenes and the abundance of each unigene in different libraries was estimated using the RSEM software [27]. The expected count data produced by RSEM (Additional file 1: Table S5) was used to identify DEGs with DEseq2 software [28]. To test reproducibility among two biological replicates, a Multi-Dimensional Scaling (MDS) plot (Fig. 3) was generated for the control and salt-treated samples of Supreme and Parish. The fact that our biological replicates cluster so closely to each other on an ordination plot demonstrates their low inter-sample variability. Two comparisons were conducted: salt-treated Supreme versus untreated Supreme and salt-treated Parish versus untreated Parish. As shown in Fig. 4a, a total of 828 unigenes were differentially expressed for salt-treated Supreme while 2222 unigenes were differentially expressed for salt-treated Parish. 34 and 107 DEGs were identified to be potential transcription factors for Supreme and Parish, respectively (Fig. 4b). Overlapping of two DEG lists generates 231 unigenes, out of which 12 unigenes are potential transcription factors (Fig. 4a and b). The commonly regulated transcription factors in both cultivars under salt treatment are listed in Additional file 1: Table S6.

### Gene enrichment analysis of DEGs identified in supreme and parish under salt treatment

**Fig. 5:**
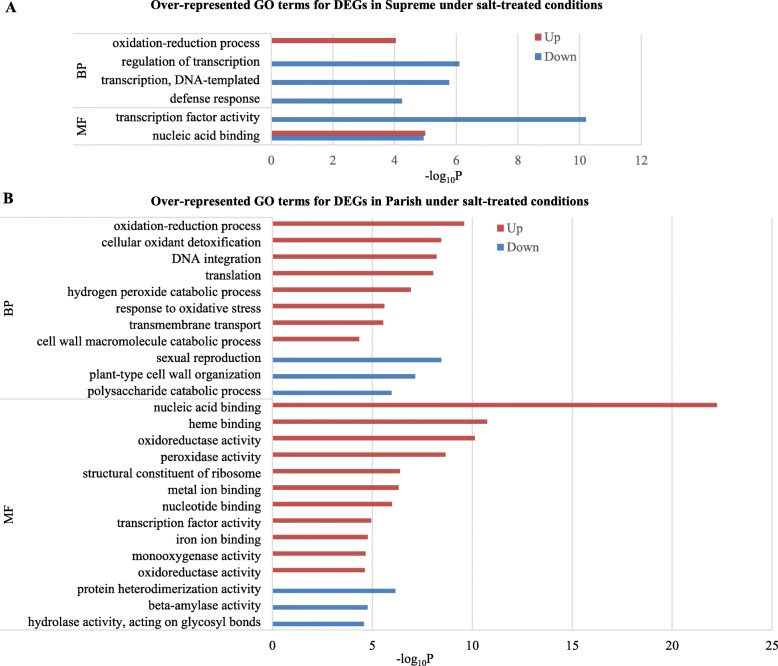
Functional enrichment analysis for DEGs identified in salt-treated (**a**) Supreme and (**b**) Parish, respectively. The y-axis shows significantly enriched gene ontology (GO) terms (*P* ≤ 0.05, Bonferroni ≤0.05) in two categories, Biological Process (BP) and Molecular Function (MF). The x-axis shows the –log_10_*P* values of these terms. Red bars, up-regulated genes; blue bars, down-regulated genes

To inspect the biological relevance of DEGs, GO terms were assigned using Blast2GO. Five-hundred out of 828 DEGs (60.4%) were annotated for Supreme while 1271 out of 2222 DEGs (57.2%) were annotated for Parish (Fig. 4a). GO enrichment analysis was then conducted to extract the over-represented GO terms that are significantly associated with the identified DEGs in Supreme and Parish under salt treatment, respectively. As shown in Fig. 5a, genes that are up-regulated in salt-treated Supreme are involved in “oxidation-reduction process” and “nucleic acid binding” while genes that are down-regulated in salt-treated Supreme are involved in “regulation of transcription”, “transcription, DNA-templated”, “defense response” and “transcription factor activity”. GO functional enrichment analysis of DEGs in salt-treated Parish revealed that they are involved in much broader processes (Fig. 5b). Many biological processes that are associated with salt response are induced in Parish, such as “oxidation-reduction process”, “cellular oxidant detoxification”, “response to oxidative stress”. Interestingly, “oxidation-reduction process” and “nucleic acid binding” are the most significantly enriched GO terms in the Biological Process (BP) category and Molecular Function (MF) category, respectively for up-regulated genes in both Supreme and Parish, implying their importance in salt tolerance in both cultivars. DEGs involved in “oxidation-reduction process” and “nucleic acid binding” are listed in Additional file 1: Table S7 and S8, respectively.

### Salt stress induced genes show higher expression in supreme than in parish under normal conditions

**Fig. 6:**
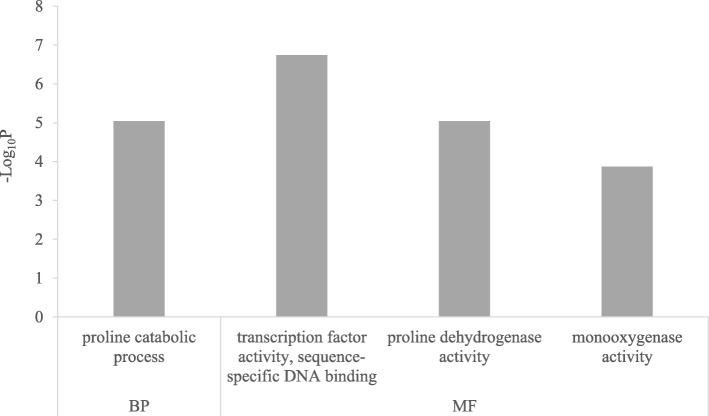
Functional enrichment analysis for salt-induced genes that show higher expression in Supreme than in Parish under normal conditions. The x-axis shows significantly enriched gene ontology (GO) terms (*P* < 0.05, Bonferroni < 0.05) in two categories, Biological Process (BP) and Molecular Function (MF). The y-axis shows the –log_10_P values of these terms

Although Supreme has fewer genes that are responsive to salt treatment than Parish, Supreme exhibits much higher tolerance than Parish. It is possible that Supreme may have a higher expression of salt stress induced genes than Parish under normal conditions that may or may not be induced upon salt treatment, and therefore may be more prepared when exposed to salinity. To test this hypothesis, we selected 202 genes based on the following criteria: 1) salt-induced genes in Parish; 2) higher expression in Supreme than in Parish under normal condition; 3) not changed or further induced in Supreme under salt treatment. To get insight into the biological meanings of these genes, we conducted GO enrichment analysis and found the following over-represented GO terms: “proline catabolic process”, “transcription factor activity”, “proline dehydrogenase activity” and “monooxygenase activity” (Fig. 6). We then further examined genes with “transcription factor activity” (Table 1). It is interesting that many of these transcription factors have been associated with salt tolerance in the previous studies, such as dehydration-responsive element-binding (DREB) proteins, ethylene-responsive transcription factors (ERFs), and WRKY transcription factors [29].

**Table 1:** Summary of salt-induced transcription factors that are enriched among genes showing higher expression level in Supreme than in Parish under normal conditions

Gene_ID	Description	Log_2_FC (S_normal_/P_normal_)	Log_2_FC (S_salt_/S_normal_)	Log_2_FC (P_salt_/P_normal_)
m.108243	hypothetical protein [*Paspalum vaginatum*]	2.26	NA^a^	1.95
m.237095	hypothetical protein SORBIDRAFT_02g026630 [*Sorghum bicolor*]	2.27	NA	3.54
m.114339	hypothetical protein SORBIDRAFT_03g034670 [Sorghum bicolor]	9.36	NA	7.50
m.43990	hypothetical protein SORBIDRAFT_03g038210 [Sorghum bicolor]	1.49	NA	2.06
m.108223	hypothetical protein SORBIDRAFT_04g031960 [Sorghum bicolor]	1.97	NA	2.02
m.285764	hypothetical protein SORBIDRAFT_06g025900 [Sorghum bicolor]	3.56	NA	4.76
m.133559	PREDICTED: AP2-like ethylene-responsive transcription factor AIL5 [*Setaria italica*]	1.82	NA	1.51
m.108267	PREDICTED: dehydration-responsive element-binding protein 1A-like [Setaria italica]	1.81	NA	2.44
m.85022	PREDICTED: dehydration-responsive element-binding protein 1E [Setaria italica]	2.73	NA	3.88
m.26812	PREDICTED: dehydration-responsive element-binding protein 1H-like [Setaria italica]	2.72	1.01	4.46
m.84649	PREDICTED: ethylene-responsive transcription factor 2 [Setaria italica]	1.07	NA	1.00
m.204461	PREDICTED: ethylene-responsive transcription factor ERF027-like [Setaria italica]	1.22	NA	1.67
m.73960	PREDICTED: ethylene-responsive transcription factor ERF109-like [Setaria italica]	1.84	NA	3.03
m.195857	PREDICTED: homeobox-leucine zipper protein HOX25-like [Setaria italica]	1.30	NA	1.08
m.60871	PREDICTED: probable WRKY transcription factor 4 [Setaria italica]	1.21	NA	2.28
m.264805	PREDICTED: probable WRKY transcription factor 41 isoform X2 [*Zea mays*]	2.23	NA	1.78
m.298519	PREDICTED: probable WRKY transcription factor 70 [Setaria italica]	1.23	NA	1.28
m.160848	PREDICTED: transcription factor HBP-1b(c1)-like [Setaria italica]	1.23	NA	1.51
m.73865	PREDICTED: WRKY transcription factor 18-like [Setaria italica]	1.55	NA	3.24
m.263026	PREDICTED: zinc finger protein ZAT9 [*Brachypodium distachyon*]	1.17	NA	1.54
m.264779	TPA: putative WRKY DNA-binding domain superfamily protein [Zea mays]	1.04	NA	1.61

^a^NA not applicable. Expression change that didn’t pass the DEGs analysis statistics (2-fold change or above, and an adjusted *P* value ≤0.01) is annotated as NA

### Genes encoding for vacuolar Na^+^/H^+^ antiporters and proton pumps are differentially expressed between supreme and parish

As Supreme accumulated more Na^+^ and showed higher salt tolerance than Parish, we speculated that the former may have developed a strong capacity to sequestrate excessive Na^+^ into the vacuole through vacuolar Na^+^/H^+^ antiporters, thus maintaining high osmotic pressure to facilitate water uptake and protecting the cytoplasm from Na^+^ toxicity. To this end, we identified a total of seven candidate Na^+^/H^+^ antiporters (m.194123, m.133530, m.194121, m.194125, m.207121, m.28253, m.170234) in seashore paspalum’s transcriptome (Table 2). The differentially expressed Na^+^/H^+^ antiporter genes are highlighted in bold font, one of which, m.194123 exhibits much higher expression in Supreme than in Parish under both normal and salt treated conditions. Interestingly, this gene is not induced by salt treatment in both Supreme and Parish. Among the remaining two differentially expressed candidate Na^+^/H^+^ antiporter genes, m.194121 has higher expression in Parish than in Supreme under salt treated conditions while m.170234 exhibits higher expression in Parish than in Supreme under normal conditions.

**Table 2:** Summary of possible Na^+^/H^+^ antiporters in seashore paspalum’s transcriptome and their expression change under different conditions. DEGs (2-fold change or above, and an adjusted *P* value ≤0.01) are in bold font

Gene_ID	Description	Log_2_FC (S_normal_/P_normal_)	Log_2_FC (S_salt_/S_normal_)	Log_2_FC (P_salt_/P_normal_)	Log_2_FC (S_salt_/P_salt_)
m.194123	PREDICTED: sodium/hydrogen exchanger 2-like [Setaria italica]	**8.88**	−0.22	−1.09	**9.74**
m.133530	sodium/hydrogen exchanger [Zea mays]	0.49	0.02	−0.07	0.58
m.194121	PREDICTED: sodium/hydrogen exchanger 2-like [Setaria italica]	−0.01	− 0.97	0.17	**−1.15**
m.194125	PREDICTED: sodium/hydrogen exchanger 2-like [Setaria italica]	0.25	−0.43	0.3	−0.49
m.207121	PREDICTED: sodium/hydrogen exchanger 6-like [Setaria italica]	0.55	−0.1	−0.1	0.55
m.28253	PREDICTED: sodium/hydrogen exchanger 8 [Setaria italica]	0.52	0.09	−0.3	0.92
m.170234	PREDICTED: sodium/hydrogen exchanger 2 [Setaria italica]	**−1.1**	−0.1	−0.37	− 0.83

As vacuolar Na^+^/H^+^ antiporters are empowered by the electrochemical gradient created by H^+^-ATPases and H^+^-pyrophosphatases (H^+^-PPases) [30], we also identified eleven H^+^-ATPases and four H^+^-PPases in seashore paspalum’s transcriptome, which are shown in Table 3 and Table 4, respectively. None of the H^+^-ATPases showed differential expression (Table 3). Interestingly, all of the four vacuolar H^+^-PPases showed lower expression level in Supreme than in Parish under normal conditions, especially for one of the vacuolar H^+^-PPase m.112845 (Table 4). However, m.112845 was induced by about 1024 times (FC = 2^10.28^) in Supreme under salt treatment, suggesting a possible role in facilitating Na^+^ sequestration under high salinity and conferring salinity tolerance in Supreme (Table 4).

**Table 3:** Summary of possible vacuolar H^+^- ATPases in seashore paspalum’s transcriptome and their expression change under different conditions. Note that vacuolar H^+^- ATPases are not differentially expressed for different comparisons indicated below

Gene_ID	Description	Log_2_FC (S_normal_/P_normal_)	Log_2_FC (S_salt_/S_normal_)	Log_2_FC (P_salt_/P_normal_)	Log_2_FC (S_salt_P_salt_)
m.102654	PREDICTED: V-type proton ATPase catalytic subunit A [Brachypodium distachyon]	0.16	0.46	−0.07	0.69
m.116106	PREDICTED: V-type proton ATPase subunit F-like [Setaria italica]	−0.22	0.04	0.15	−0.33
m.117254	Vacuolar proton pump 16 kDa proteolipid subunit	−0.23	0.15	−0.08	−0.01
m.117255	PREDICTED: V-type proton ATPase 16 kDa proteolipid subunit [Oryza brachyantha]	−0.19	0.26	0.19	−0.12
m.117270	PREDICTED: V-type proton ATPase 16 kDa proteolipid subunit [Oryza brachyantha]	−0.51	0.27	0.16	−0.39
m.173282	PREDICTED: V-type proton ATPase subunit a1 [Setaria italica]	0.21	0.06	0.02	0.25
m.190922	PREDICTED: V-type proton ATPase subunit E [Setaria italica]	−0.73	0.47	0.68	−0.94
m.23021	putative ATPase, V1 complex, subunit A protein [Zea mays]	0.29	−0.08	0.34	−0.12
m.230918	PREDICTED: V-type proton ATPase subunit G1-like [Oryza brachyantha]	−0.58	0.13	0.18	−0.62
m.232963	PREDICTED: V-type proton ATPase subunit a3-like [Setaria italica]	−0.38	0.24	0.17	−0.32
m.279500	V-type proton ATPase subunit E-like [Zea mays]	−0.27	0.20	0.12	−0.19

**Table 4:** Summary of possible vacuolar H^+^-PPases in seashore paspalum’s transcriptome and their expression change under different conditions. DEGs (2-fold change or above, and an adjusted P value ≤0.01) are in bold font

Gene_ID	Description	Log_2_FC (S_normal_/P_normal_)	Log_2_FC (S_salt_/S_normal_)	Log_2_FC (P_salt_/P_normal_)	Log_2_FC (S_salt_P_salt_)
m.112845	V-type H(+)-translocating pyrophosphatase [Aphanomyces invadans]	**−8.48**	**10.28**	0.69	**1.12**
m.73322	PREDICTED: pyrophosphate-energized vacuolar membrane proton pump-like [Setaria italica]	**−1.68**	−0.94	0.30	**−2.92**
m.88459	PREDICTED: pyrophosphate-energized vacuolar membrane proton pump-like [Setaria italica]	**−1.83**	**− 1.16**	0.30	**−3.29**
m.95345	PREDICTED: pyrophosphate-energized vacuolar membrane proton pump-like isoform X1 [Setaria italica]	**−2.20**	**1.11**	0.43	**−1.52**

## Discussion

### Supreme takes advantage of Na^+^ accumulation for improved salt tolerance

It becomes evident that the mechanisms that contribute to high salt-tolerance in halophytes are conserved to those known in glycophytes, the plant species susceptible to salinity although some halophytes have evolved special adaptive mechanisms such as salt glands to actively excrete salts [31]. However, halophytes may possess unique genomic structure (e.g. a higher gene copy number and altered promoter sequences), and subtle gene regulation at the transcription and protein levels that leads to their better adaption to high salinity in the environment [32].

In our study, we investigated the mechanisms underlying salt tolerance in a halophyte called seashore paspalum by comparing two cultivars: Supreme (high salt-tolerance) and Parish (moderate salt-tolerance) at physiological and transcriptome levels under both non-treated and salt-treated conditions (400 mM NaCl). Measurement of Na^+^ content suggests that Na^+^ accumulation under both normal and salt-treated conditions is a key mechanism underlying Supreme’s high salinity tolerance. Na^+^ accumulation by Supreme under salt treatment is not surprising as previous studies suggest that this is a common mechanism for both halophytes, the salt-tolerant plants and glycophytes, the plant species susceptible to salinity under salt stress to facilitate water uptake [33]. However, the seashore paspalum genotype, Supreme takes full advantage of this mechanism by accumulating Na^+^ in a significantly higher level than Parish under normal conditions, which may be evolved as a protective mechanism for osmotic adjustment to counteract high levels of Na^+^ in the surrounding environment.

We suggest that further increased Na^+^ in Supreme under salt-treated conditions is sequestrated into the vacuole to prevent its toxicity to the cytoplasm. Na^+^ sequestration into the vacuole takes place by the operation of vacuolar Na^+^/H^+^ antiporters (NHXs) in concert with two proton pumps H^+^-ATPases and H^+^-PPases. Genes involved in Na^+^ sequestration are promising candidate genes to engineer crops for salinity tolerance. Several salinity tolerant plants have been successfully developed by overexpression of either NHXs or H^+^-PPases (e.g. AVP1) [30]. In our study, we identified at least two possible vacuolar Na^+^/H^+^ antiporters (NHXs), namely m.133530 and m.170234 (Table 2). Of the remaining five NHXs, m.194123 exhibits dramatically higher expression in Supreme than in Parish under both normal and salt-treated conditions, raising the question of whether or not m.194123 functions as a vacuolar Na^+^/H^+^ antiporter. We also identified four H^+^-PPases, namely m.112845, m.73322, m.88459 and m.95345, of which m.112845 was highly induced by salt treatment in Supreme despite its lower expression than Parish under normal conditions (Table 4). The function and activity of these NHXs and H^+^-PPases are all worth further examination.

### Elevated expression of salt stress induced transcription factors in supreme under normal conditions, possibly due to enhanced Ca^2+^ signaling, is another contributor to Supreme’s higher salt tolerance

As a terminal transducer of the salt stress signaling pathway, transcription factors (TFs) can directly regulate the expression of an array of downstream stress-responsive genes through interaction with the specific cis-acting elements in their promoter region. In our study, we found that an array of salt stress induced transcription factors showed higher expression level in Supreme than in Parish under normal conditions (Table 1). Some of these transcription factors are associated with salt stress response, including dehydration-responsive element-binding (DREB) proteins, ethylene-responsive transcription factors and WRKY transcription factors [29]. This result is consistent with previous study of transcriptomic variation of three different ecotypes of *Arabidopsis* (Col, Ler, and Sha) in response to salt stress, in which it was found that there existed extensive differences in gene expression between the salt-tolerant ecotype Sha and the other two relatively salt-sensitive ecotypes Col and Ler for salt stress related TFs, such as heat shock TFs (HSF) under normal conditions [34]. It is possible that the elevated expression of salt stress induced TFs in Supreme under normal conditions contributes to its higher salt-tolerance and this mechanism may be conserved between different salt-tolerant plant species.

Ca^2+^ is a very important second messenger in response to a wide range of external stimuli, including salt stress. High salinity causes a rapid and transient increase in cytosolic Ca^2+^, which is further decoded by Calcineurin B-like protein (CBL)-CBL-interacting protein kinase (CIPK) complex to initiate a phosphorylation/dephosphorylation cascade, resulting in regulation of multiple stress-responsive genes and ultimately leading to phenotypic response of stress tolerance directly or indirectly [35]. Higher Ca^2+^ accumulation in Supreme (possibly triggered by Na^+^ accumulation) than in Parish under normal conditions may account for the elevated expression of salt stress responsive TFs in Supreme through high Na^+^-triggered Ca^2+^ signaling pathway (Fig. 1f). Supporting this hypothesis, salt-treated Parish accumulated Na^+^ and Ca^2+^ to a level that is comparable to the Na^+^ and Ca^2+^ content in non-treated Supreme, which coincides with the induction of many salt stress responsive TFs.

### Intracellular K^+^ retention under high salinity may contribute to salinity tolerance in both cultivars

K^+^ uptake at the root-soil interface is mainly mediated by high affinity uptake transporters (μM range) and low affinity uptake transporters (mM range). While the former uptake mechanism is performed by members of the KT/HAK/KUP family such as high affinity potassium transporter 5 (HAK5) and potassium uptake transporter 7 (KUP7), the latter uptake mechanism is achieved by K^+^ channels of the Shaker family, such as Arabidopsis K^+^ transporter (AKT1) [36]. Xylem K^+^ loading from the root is carried out by stelar K^+^ outward rectifying channels (SKORs) and KUP7 in *Arabidopsis* [37] while K^+^ transport across the vascular bundle to mesophyll cells in the shoot has not been clearly elucidated so far. Under salt stress, high levels of Na^+^ often inhibit K^+^ uptake and induce K^+^ efflux in both root and leaf cells due to Na^+^-induced plasma membrane (PM) depolarization and a consequential inhibition of K^+^ uptake channels and activation of K^+^ efflux channels such as K^+^ outward rectifying channels (KORs) and nonselective cation channels (NSCCs). Thus, K^+^ deficiency often occurs under salt stress, which results in growth inhibition [36, 38]. The capacity to retain intracellular K^+^, which counteracts the toxic effect of excessive Na^+^, was regarded as equally important mechanism to the regulation of toxic Na^+^ accumulation for salt stress tolerance [39]. In our study, both Supreme and Parish maintained a stable K^+^ level after salt treatment, suggesting that K^+^ retention, possibly by maintaining negative membrane potential may play a critical role for salinity tolerance in both cultivars. An important question to be addressed in the future is how Supreme and Parish alleviate Na^+^-induced PM depolarization to maintain negative membrane potential for K^+^ retention under salt conditions. Moreover, we identified a total of 18 putative potassium transporters in seashore paspalum’s transcriptome, of which m.149226 is a high affinity potassium transporter and m.6215 is a predicted low affinity uptake channel AKT2 (Table 5). Further characterization of these potassium transporter genes would shed light on their roles in potassium uptake and translocation.

**Table 5:** Summary of possible K^+^ transporters in seashore paspalum’s transcriptome and their expression change under different conditions. DEGs (2-fold change or above, and an adjusted P value ≤0.01) are in bold font

Gene_ID	Description	Log_2_FC (S_normal_/P_normal_)	Log_2_FC (S_salt_/S_normal_)	Log_2_FC (P_salt_/P_normal_)	Log_2_FC (S_salt_/P_salt_)
m.124553	PREDICTED: potassium transporter 10-like [Setaria italica]	0.56	0.00	0.82	−0.27
m.149226*	high-affinity potassium transporter [*Phragmites australis*]	−0.85	**2.86**	1.26	0.75
m.167648	PREDICTED: potassium channel KOR1 [Setaria italica]	**−1.29**	**1.39**	**1.27**	**−1.17**
m.169812	potassium transporter [Phragmites australis]	**−1.12**	−0.28	0.19	**−1.59**
m.169813	potassium transporter [Phragmites australis]	0.97	−0.85	−0.24	0.36
m.177897	PREDICTED: potassium transporter 1-like [Setaria italica]	0.00	**2.08**	0.86	**1.23**
m.210030	PREDICTED: potassium transporter 25 [Setaria italica]	**−1.54**	−0.46	−0.12	**−1.88**
m.222898	Putative potassium transporter 14 [*Aegilops tauschii*]	−0.86	−0.08	0.21	**−1.15**
m.259914	PREDICTED: two-pore potassium channel 2-like [Setaria italica]	−1.47	0.50	−0.70	−0.28
m.261833	potassium channel [Saccharum hybrid cultivar]	**1.32**	−0.35	0.65	0.32
m.268433	PREDICTED: probable potassium transporter 11 [Setaria italica]	−1.16	−0.26	0.41	**−1.82**
m.307318	potassium transporter [Phragmites australis]	0.06	−0.37	0.26	−0.57
m.307324	PREDICTED: probable potassium transporter 9 [Setaria italica]	**1.08**	0.31	**2.00**	−0.62
m.58659	PREDICTED: probable potassium transporter 11 [Setaria italica]	−0.66	0.10	−0.06	−0.49
m.5987	PREDICTED: potassium transporter 22-like [Setaria italica]	−0.25	−0.75	− 0.12	−0.87
m.6215*	PREDICTED: potassium channel AKT2 [Setaria italica]	1.10	−0.38	0.45	0.27
m.77121	PREDICTED: potassium transporter 24-like [Setaria italica]	0.04	−0.13	0.26	−0.35
m.79462	PREDICTED: probable potassium transporter 16 [Setaria italica]	**−1.82**	0.43	0.08	**−1.48**

### Oxidation-reduction regulation and nucleic acid binding activity under high salinity may be other important factors for salinity tolerance in both cultivars

Salt stress can lead to the accumulation of ROS, causing oxidative stress to the plants. The oxidation-reduction process is critical for salinity tolerance in plants as it is involved in scavenging ROS and maintaining oxidation-reduction homeostasis. In our study, “oxidation-reduction process” is the most significantly enriched GO term in the BP category for both Supreme and Parish up-regulated genes under salt treatment (Fig. 5), which indicates that this process may play an important role in salt tolerance in both cultivars. This result is consistent with previous transcriptome profiling study in a halophyte, ice plant (*Mesembryanthemum crystallinum*) under high salinity, suggesting that oxidation-reduction may be a conserved mechanism conveying salt tolerance [40]. Accordingly, several oxidoreductase genes such as glutathione-disulfide reductase (GSR), superoxide dismutase (SOD), aldehyde dehydrogenase (ALDHs), and peroxidases are upregulated in Supreme (Additional file 1: Table S7A) while more oxidoreductase genes including ALDHs and peroxidases are upregulated in Parish under salt treatment (Additional file 1: Table S7B).

“Nucleic acid binding” is the most significantly enriched GO term in the MF category for both Supreme and Parish up-regulated genes under salt treatment, suggesting that this process may also play a crucial role in salt tolerance in both cultivars. In Supreme, a DEAD-box ATP-dependent RNA helicase gene (m.319487) was upregulated over 100-fold (FC = 2^6.92^) under high salinity conditions (Additional file 1: Table S8A), implying a possible role in salinity tolerance. DEAD-box RNA helicases are regarded as RNA chaperones as these proteins can unwind misfolded RNAs with non-functional secondary structures for correct folding using energy derived from ATP hydrolysis, ensuring the translation initiation inhibited by stress to proceed [10, 41]. Overexpression of an *Apocynum venetum* DEAD-box helicase 1 (*AvDH1*) in cotton under CaMV 35S promoter confers salinity tolerance and increasing crop productivity in saline fields [42]. Expression of a putative DEAD-Box RNA helicase gene *SlDEAD31* in tomato was induced by heat, cold, and dehydration. Transgenic tomato plants overexpressing *SlDEAD31* exhibited significantly improved salt tolerance and slightly improved drought resistance compared to wild-type plants [43]. It will be interesting to overexpress the salt stress induced DEAD-box RNA helicase gene identified in Supreme in model species such as *Arabidopsis* to test whether it confers salinity tolerance.

## Conclusions

**Fig. 7:**
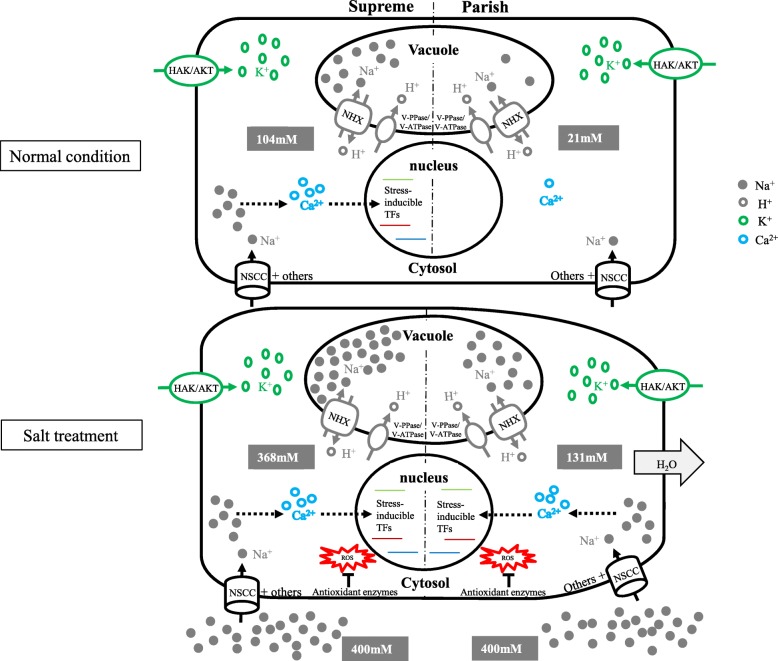
A schematic model for the salinity tolerance mechanisms in Supreme versus the salinity tolerance mechanisms in Parish. Numbers indicated are intracellular and extracellular Na^+^ concentrations. ROS detoxification and maintaining K^+^ uptake under salt stress are two common mechanisms for salinity tolerance in both cultivars. High Na^+^ levels in Supreme under normal and salt-treated conditions lower the water potential, preventing water loss. Moreover, an array of salt stress inducible transcription factors is highly expressed in Supreme under normal conditions, possibly induced by the Ca^2+^ signaling pathway due to Na^+^ accumulation under normal conditions, making Supreme prepared for salt stress

Based on our results, we proposed a hypothetical model depicting the mechanisms underlying Supreme’s high salt tolerance (Fig. 7). We suggest that Na^+^ accumulation under normal conditions and the resulting osmotic adjustment and the expression of salt stress responsive transcription factors induced by Ca^2+^ signaling pathway, possibly due to Na^+^ accumulation under normal conditions, are two important protective mechanisms that are responsible for the higher salinity tolerance observed in Supreme. In addition, K^+^ retention, strong oxidation-reduction processes, and nucleic acid binding activities under high salinity conditions may also contribute to the salinity tolerance in both cultivars. Ion transporters, including NHXs coupled with H^+^-PPases and K^+^ uptake transporters, salt stress responsive transcription factors, oxidoreductases and the salt stress induced DEAD-box RNA helicase identified in Supreme in this study can be used as candidate genes for functional studies and potential targets to engineer plants for enhanced salinity tolerance, opening new avenues for future research. It should be noted that given the limited sampling time points and biological replicates for transcriptome analysis in the current study, more comprehensive research in the future would further our understanding of the molecular mechanisms underlying the high salt tolerance in *Paspalum vaginatum*.

## Methods

### Plant materials growth and treatment

Two cultivars of seashore paspalum, Supreme and Parish obtained from Department of Crop & Soil Sciences, University of Georgia were clonally propagated from the same number of tillers in pure sand for 8 weeks in 10 × 10 cm square containers. They were maintained in the growth room under 14 h of photoperiod with 350 to 450 μmol m^− 2^ s^− 1^ illumination. Temperature and humidity were maintained at 25 °C and 30% during the daytime and 17 °C and 60% at night. For the morphological observation of plant performance under salt stress, Supreme and Parish were immersed in a 400 mM NaCl solution supplemented with 0.2 g/l water soluble fertilizer (20:10:20 nitrogen:phosphorus:potassium; Scotts). Twelve days after salt treatment, plants were recovered from salt stress by washing off NaCl and watering with 0.2 g/l water soluble fertilizer every other day. Plants were photographed 8 days after recovery for documentation. To collect salt-treated samples for RNA-seq, salt treatment was performed by washing the sand off roots and dipping them in 400 mM NaCl solution supplemented with 0.2 g/l water soluble fertilizer for 1 h.

### Measurement of Na^+^, K^+^ and Ca^2+^ content

For Na^+^, K^+^ and Ca^2+^ content measurements, three biological replicates of the leave samples from Supreme and Parish were collected before and after a 7-day treatment of 400 mM NaCl solution supplemented with 0.2 g/l water soluble fertilizer, and then dried for 48 h at 80 °C. Na^+^, K^+^ and Ca^2+^ from the whole leaf were extracted using the modified Kjeldahl procedures and measured using inductively coupled plasma (ICP)-atomic emission spectrometry based on previous protocols [44, 45].

### RNA isolation and cDNA library preparation

One hundred milligrams of mixed tissue (leaf:stem:root =1:1:1) was collected immediately after treatment and ground into a fine powder for RNA exaction using Trizol (Invitrogen) following the manufacturer’s protocol. Total RNA was then treated with DNase to eliminate DNA contamination and purified using the RNeasy Mini Kit (Qiagen). Total RNA fractions with 260/280 absorbance of 2.0 and RNA integrity of 8.0 or higher were used for further experiments. cDNAs were then synthesized for RNA-seq library construction using the Illumina TruSeq® RNA Sample Preparation Kit with Oligo-dT beads capturing polyA tails. Eight cDNA libraries were constructed, which were divided into 4 groups with each of the group having two biological replicates: untreated Supreme (S_normal_-1, S_normal_-2), salt-treated Supreme (S_salt_-1, S_salt_-2), untreated Parish (P_normal_-1, P_normal_-2), and salt-treated Parish (P_salt_-1, P_salt_-2). RNA extraction and an additional 4 cDNA libraries were also constructed for drought-treated Supreme (S_drought_-1, S_drought_-2) and drought-treated Parish (P_drought_-1, P_drought_-2). The reads generated from these drought-treated samples were included in the de novo transcriptome assembly to increase assembly continuity but were not used for other analyses in this paper.

### Transcriptome sequencing and de novo assembly

Paired-end sequencing of cDNA libraries was performed using the HiSeq 2000 (Illumina Technologies) platform. The raw reads were evaluated for quality using FastQC (version: 0.11.3, http://www.bioinformatics.babraham.ac.uk/projects/fastqc/), and then trimmed to remove adapter sequences and low quality bases using Trimmomatic 0.32 [46]. The trimmed reads were used to generate a de novo assembly using Trinity (version: trinityRNA-seq-2.1.1) with default k-mer length of 25 [47]. The RNA-seq reads with quality scores were deposited in the NCBI Sequence Read Archive (SRA) with bioproject accession number PRJNA395934.

### ORF identification and sequence annotation

The next step in the pipeline is to identify potential protein coding genes by using TransDecoder (version: TransDecoder-2.0, http://transdecoder.github.io/). 169,391 ORFs (49.5% of all Trinity transcripts) were identified among 342,165 Trinity transcript sequences using TransDecoder based on the following criteria: a minimum length of 100 amino score and greater than 0 is reported; if a shorter ORF is fully encapsulated by a longer ORF, the longer one is reported; any ORF that does not meet the above criteria but has homology to the UniProt and Protein family (Pfam) databases will also be retained. CD-HIT (version: cd-hit-v4.6.6) [48] clustered the remaining genes with a sequence identity ≥95%. This generated a final set of 82,608 potential protein coding unigenes. The Benchmarking Universal Single Copy Orthologs (BUSCO) (version: BUSCO 3.0.1) software was used to validate the completeness of the assembled transcriptome [49]. To obtain sequence annotation, they were blasted against the NCBI non-redundant (nr) protein database by using NCBI-BLAST+ (version: ncbi-blast-2.3.0+) [50] with an E-value cutoff of 1E^− 5^ and putative GO terms were assigned by running Blast2GO software (version 3.3) [24]. Unigenes were blasted against the plant transcription factor database (PlantTFDB) [25, 26] (http://planttfdb.cbi.pku.edu.cn/index.php?sp=Ath) with E-value cutoff of 1E^− 5^ to identify transcription factors in seashore paspalum’s transcriptome. The blast results were then parsed by a Python script to count the number of unigenes that have at least one hit to the putative transcription factors of *Arabidopsis* and *Oryza* in different transcription factor families.

### Differential expression analysis

To identify differentially expressed genes, the trimmed reads from each sample were aligned to the 82,608 reference unigenes and an abundance estimation for each unigene in each sample was then calculated with RSEM software (version: RSEM-1.2.28) [27]. The MDS plot was generated by using the expected counts generated by RSEM to ordinate samples in multidimensional space based on differences in expression values. The percentage of variance in each of the MDS axes was calculated using the Bioconductor package Glimma [51]. The expected counts generated by RSEM were then used as input for differential expression analysis using DEseq2 software [28]. Four comparisons were conducted: 1) untreated Supreme (Snormal) versus untreated Parish (Pnormal), 2) salt-treated Supreme (Ssalt) versus untreated Supreme (S_normal_), 3) salt-treated Parish (P_salt_) versus untreated Parish (P_normal_), and 4) salt-treated Supreme (S_salt_) versus salt-treated Parish (P_salt_). Differentially expressed genes are defined by a log_2_ fold change (FC) ≥ 1.0 or ≤ − 1.0, and an adjusted *P* value ≤0.01. To determine the differentially expressed transcription factors, the generated lists of DEGs were overlapped with the potential transcription factors identified in seashore paspalum’s transcriptome described above using a R script, and where they intersected defined the differentially expressed transcription factors.

### GO enrichment analysis

Given that seashore paspalum does not have an official ontology, a custom annotation list was generated as described above. To find significantly enriched GO terms, we calculated the P value from a Fisher’s exact test between the frequency of the GO terms for genes in the differentially expressed set and the custom annotation serving as our background by using a scipy.stats package in a Python script [52]. The P value threshold was set as *P* ≤ 0.05. To account for multiple testing, we adjusted the *P* values using a R script and used the Bonferroni value ≤0.05*.*

## Supplementary information

**Additional file 1: Figure S1.** Size distribution of unigenes. **Figure S2.** E-value distribution of the Blastx hits against the nr protein database with an E-value cutoff of 1E^− 5^. **Figure S3.** Pie chart representation of seashore paspalum’s transcriptome GO annotation on level 2. **Figure S4.** Species distribution of unigenes. **Table S1.** Summary of transcriptome sequencing and de novo assembly. **Table S2.** Summary of annotation statistics of seashore paspalum’s transcriptome. **Table S3.** BUSCO analysis for the assessment of transcriptome completeness. **Table S4.** Transcription factors of different families in seashore paspalum’s transcriptome. **Table S6.** Summary of possible transcription factors that are commonly regulated by Supreme and Parish under salt-treated conditions. **Table S7A.** DEGs involved in “oxidation-reduction process” in salt-treated Supreme. **Table S8A.** DEGs with “nucleic acid binding activity” in salt-treated Supreme

## Data Availability

The RNA-seq datasets supporting the conclusions of this article are available in the NCBI Sequence Read Archive (SRA) with bioproject accession number PRJNA395934.

## Abbreviations

AA: Ascorbic acid
AKT1: Arabidopsis K^+^ transporter
ALDHs: Aldehyde dehydrogenase
APX: Ascorbate peroxidase
*AvDH1*: *Apocynum venetum* DEAD-box helicase 1
BP: Biological process
CAT: Catalase
CBL: Calcineurin B-like protein
CC: Cellular component
CIPK: CBL-interacting protein kinase
DHAR: Dehydroascorbate reductase
DREB proteins: Dehydration-responsive element-binding proteins
ERFs: Ethylene-responsive transcription factors
GO: Gene ontology
GPX: Glutathione peroxidase
GSH: Glutathione
GSR: Glutathione-disulfide reductase
H^+^-PPases: H^+^-pyrophosphatases
H_2_O_2_: Hydrogen peroxide
HAK5: High affinity potassium transporter 5
HSFs: Heat shock transcription factors
KORs: K^+^ outward rectifying channels
KUP7: Potassium uptake transporter 7
LEAs: Late embryogenesis abundant proteins
MF: Molecular function
NHXs: Vacuolar Na^+^/H^+^ antiporters
nr protein database: Non-redundant protein database
NSCCs: Nonselective cation channels
O_2_˙^−^: Superoxide radical anion
OH˙: Hydroxyl radicals
ORFs: Open reading frames
P_drought_: Drought-treated Parish
Pfam database: Protein family database
PM: Plasma membrane
P_normal_: Untreated Parish
P_salt_: Salt-treated Parish
ROS: Reactive oxygen species
S_drought_: Drought-treated Supreme
SKORs: Stelar K^+^ outward rectifying channels
S_normal_: Untreated Supreme
SOD: Superoxide dismutase
SOS1: Na^+^/H^+^ transporter Salt Overly Sensitive 1
SRA: Sequence Read Archive
S_salt_: Salt-treated Supreme
TFs: Transcription factors
