# Supplementary material for: Comparative transcriptome profiling provides insights into plant salt tolerance in seashore paspalum (Paspalum vaginatum)
Source: BMC Genomics. 2020 Feb 7;21:131. doi: 10.1186/s12864-020-6508-1 (PMC7006205; doi:10.1186/s12864-020-6508-1)
Supplement: Supplementary file 1 — Additional file 1: Figure S1. Size distribution of unigenes. Figure S2. E-value distribution of the Blastx hits against the nr protein database with an E-value cutoff of 1E− 5. Figure S3. Pie chart representation of seashore paspalum’s transcriptome GO annotation on level 2. Figure S4. Species distribution of unigenes. Table S1. Summary of transcriptome sequencing and de novo assembly. Table S2. Summary of annotation statistics of seashore paspalum’s transcriptome. Table S3. BUSCO analysis for the assessment of transcriptome completeness. Table S4. Transcription factors of different families in seashore paspalum’s transcriptome. Table S6. Summary of possible transcription factors that are commonly regulated by Supreme and Parish under salt-treated conditions. Table S7A. DEGs involved in “oxidation-reduction process” in salt-treated Supreme. Table S8A. DEGs with “nucleic acid binding activity” in salt-treated Supreme [file 12864_2020_6508_MOESM1_ESM.zip › 01162020_Revised Supplemental Materials.docx]

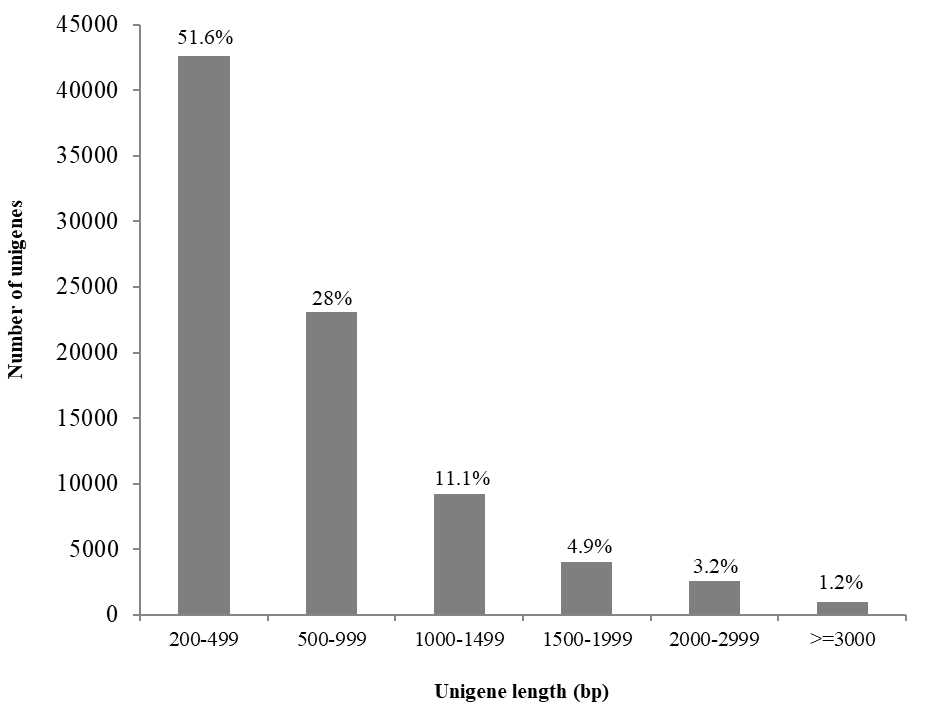


**Supplemental figure S1. Size distribution of unigenes.** Six groups of unigenes with different range of length were shown. The percentages of unigenes in each group out of the total unigenes (82,608) were indicated above each column.

**Supplemental figure S2. E-value distribution of the Blastx hits against the nr protein database with an E-value cutoff of 1E^-5^.**


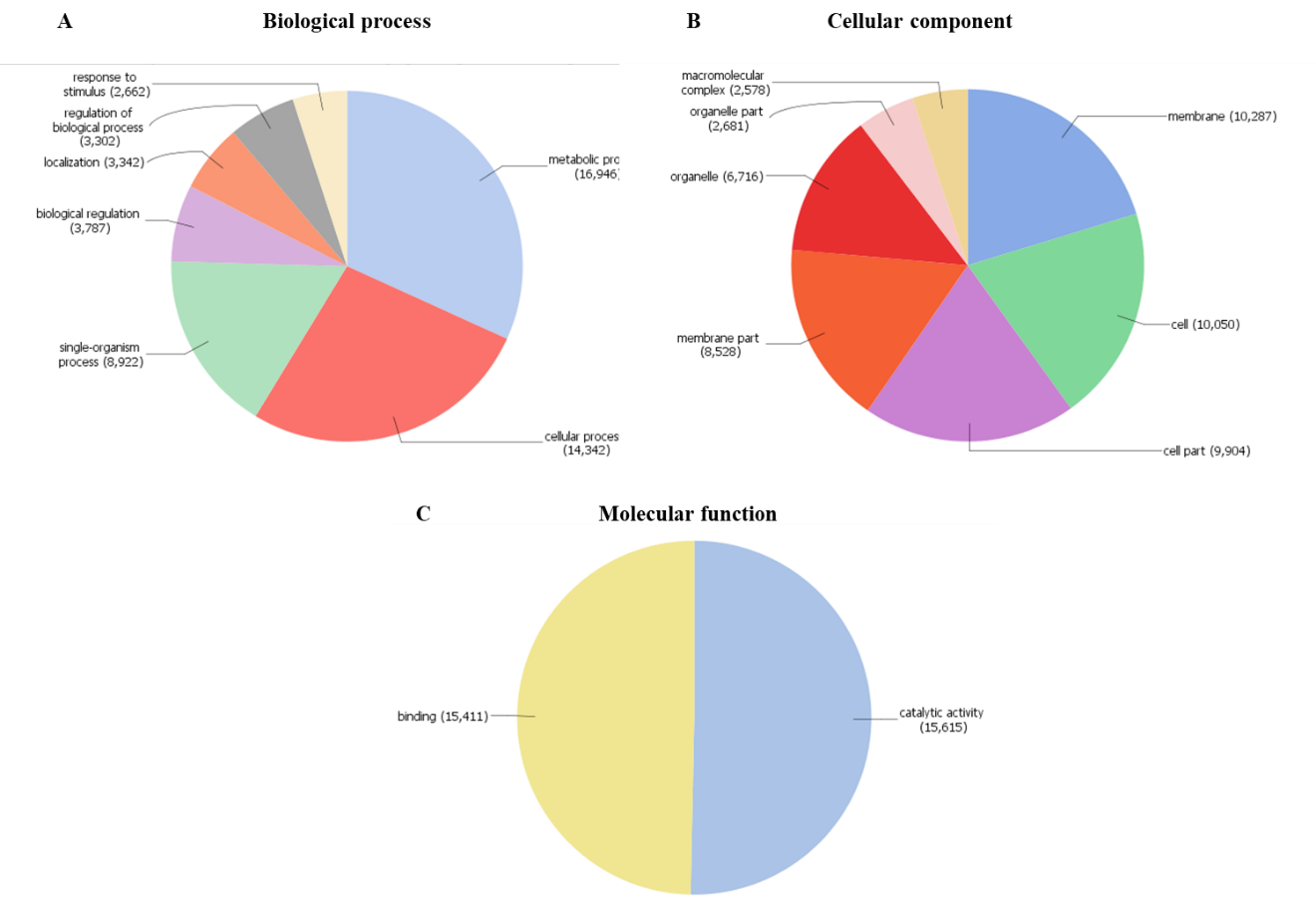


**Supplemental figure S3. Pie chart representation of seashore paspalum’s transcriptome GO annotation on level 2. (A)** Biological process; **(B)** Cellular component; **(C)** Molecular function. The number besides each GO term represents the number of sequences belonging to it.


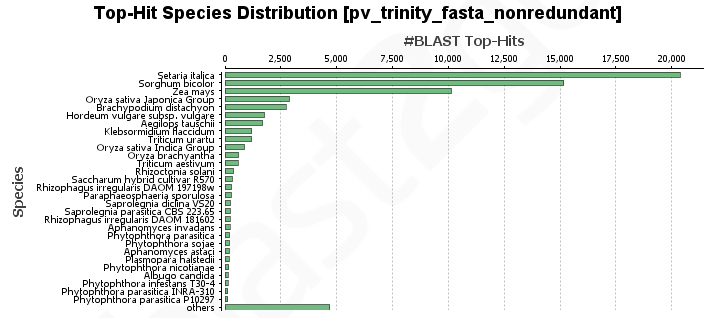


**Supplemental figure S4. Species distribution of unigenes.** Species distribution of the top blast hits for each unigene with an E-value cutoff of 1E^-5^.

**Supplemental table S1. Summary of transcriptome sequencing and de novo assembly**

| **Items** | **Supreme** | **Parish** |
| --- | --- | --- |
| Total raw reads | 80,288,751 | 78,867,558 |
| Total clean reads | 76,986,554 | 75,528,530 |
| Total Trinity transcripts | 342,165 | |
| Total Trinity genes | 244,926 | |
| Average transcript/gene length (bp) | 783.7/580 | |
| Transcript N50/gene N50 (bp)^a^ | 1,339/761 | |
| Average GC content (%)  ^a^ Transcript N50/gene N50 is defined as the length of the longest transcript/gene such that all transcripts/genes of the same or above that length compose at least 50% of the assembled base pairs. | 49.69 | |

**Supplemental table S2. Summary of annotation statistics of seashore paspalum’s transcriptome.** The assembled transcriptome was blasted against the non-redundant (nr) protein database, Interpro protein database and Plant Transcription Factor Database (PlantTFDB). Genes were assigned with gene ontology (GO) annotation by using Blast2GO software.

| **Database** | **Unigenes having homologous sequence** | |
| --- | --- | --- |
|  | **Number** | **Hit (%)** |
| nr | 65540 | 79.3% |
| Interpro | 32860 | 39.8% |
| GO | 36387 | 44% |
| PlantTFDB | 3250 | 4% |

**Supplemental table S3. BUSCO analysis for the assessment of transcriptome completeness**

|  | **Transcriptome from this manuscript** | **Transcriptome from Jia X, *et al*. Genes & genomics. 2015** |
| --- | --- | --- |
| Total BUSCO groups searched | 3,278 | 3,278 |
| Complete BUSCOs | 2,738 (92.3%) | 2,514 (76.7%) |
| Fragmented BUSCOs | 174 (5.3%) | 423 (12.9%) |
| Missing BUSCOs | 76 (2.4%) | 341 (10.4%) |

**Supplemental table S4. Transcription factors of different families in seashore paspalum's transcriptome**

| **Family** | **Number of genes** |  |
| --- | --- | --- |
| MYB family protein | 419 |  |
| WRKY family protein | 370 |  |
| G2-like family protein | 268 |  |
| bZIP family protein | 240 |  |
| bHLH family protein | 185 |  |
| C3H family protein | 175 |  |
| NAC family protein | 163 |  |
| B3 family protein | 159 |  |
| C2H2 family protein | 143 |  |
| SRS family protein | 105 |  |
| FAR1 family protein | 102 |  |
| pentatricopeptide (PPR) repeat-containing protein | 81 |  |
| Trihelix family protein | 77 |  |
| GATA family protein | 74 |  |
| protein kinase family protein | 74 |  |
| EIL family protein | 62 |  |
| ARF family protein | 47 |  |
| HB-other family protein | 41 |  |
| E2F/DP family protein | 39 |  |
| ERF family protein | 37 |  |
| GRAS family protein | 35 |  |
| HD-ZIP family protein | 33 |  |
| NF-YB family protein | 28 |  |
| LBD family protein | 26 |  |
| GRF family protein | 23 |  |
| histone-like transcription factor and archaeal histone family protein | 23 |  |
| HB-PHD family protein | 19 |  |
| CAMTA family protein | 17 |  |
| AP2 family protein | 17 |  |
| CPP family protein | 12 |  |
| LSD family protein | 11 |  |
| CO-like family protein | 10 |  |
| HSF family protein | 10 |  |
| STAT family protein | 10 |  |
| ZF-HD family protein | 9 |  |
| alpha/beta hydrolase fold, putative | 8 |  |
| BES1 family protein | 8 |  |
| WD-40 repeat family protein | 8 |  |
| MIKC family protein | 7 |  |
| Nin-like family protein | 7 |  |
| NF-YA family protein | 6 |  |
| RIPER7 - Ripening-related family protein precursor | 6 |  |
| GeBP family protein | 5 |  |
| LFY family protein | 5 |  |
| S1Fa-like family protein | 5 |  |
| TCP family transcription factor, putative | 5 |  |
| AP2 domain containing protein | 4 |  |
| NF-YC family protein | 4 |  |
| helix-loop-helix DNA-binding domain containing protein | 3 |  |
| SBP family protein | 3 |  |
| AGAMOUS-like 26 | 2 |  |
| ARR-B family protein | 2 |  |
| BEE 3, putative, expressed | 2 |  |
| Dof family protein | 2 |  |
| APRATAXIN-like | 1 |  |
| auxin response factor 19, putative | 1 |  |
| auxin response factor 9 | 1 |  |
| B-box type zinc finger protein with CCT domain | 1 |  |
| BBR-BPC family protein | 1 |  |
| CCT/B-box zinc finger protein, putative | 1 |  |
| DUF260 domain containing protein, putative | 1 |  |
| ethylene response factor 110 | 1 |  |
| homeobox associated leucine zipper, putative | 1 |  |
| no apical meristem protein, putative, expressed | 1 |  |
| nuclear transcription factor Y subunit, putative | 1 |  |
| pathogenesis related homeodomain protein A | 1 |  |
| two-component response regulator, putative | 1 |  |
| zinc finger C-x8-C-x5-C-x3-H type family protein | 1 |  |
| Total | 3250 |  |

**Supplemental table S6. Summary of possible transcription factors that are commonly regulated by Supreme and Parish under salt-treated conditions**

| **Gene_ID** | **Hit description** | **Log_2_FC**  **(S_salt_/S_normal_)** | **Log_2_FC**  **(P_salt_/P_normal_)** |
| --- | --- | --- | --- |
| m.52678 | Oryza sativa Indica Group MYB_related family protein | 2.6 | 2.2 |
| m.237571 | Oryza sativa Indica Group MYB_related family protein | 2.2 | 2.6 |
| m.48837 | Oryza barthii GATA family protein | 2.1 | 1.8 |
| m.167648 | Oryza barthii C3H family protein | 1.4 | 1.3 |
| m.88900 | Oryza sativa Indica Group WRKY family protein | 1.2 | 1.4 |
| m.96240 | Oryza barthii G2-like family protein | 1.0 | 1.0 |
| m.54046 | Oryza punctata G2-like family protein | -1.1 | -1.0 |
| m.32600 | Oryza sativa Indica Group MYB family protein | -1.1 | 1.1 |
| m.181019 | Oryza punctata bZIP family protein | -1.2 | -1.3 |
| m.65089 | Oryza sativa Japonica Group histone-like transcription factor | -1.9 | -3.0 |
| m.43705 | Arabidopsis lyrata C3H family protein | -2.5 | 1.4 |
| m.80449 | Oryza barthii SRS family protein | -2.9 | -1.7 |

**Supplemental table 7A. DEGs involved in “oxidation-reduction process” in salt-treated Supreme**

| **Gene_ID** | **Description** | **Log_2_FC** | **P value** | **Adjusted P value** |
| --- | --- | --- | --- | --- |
| **m.219752** | **alcohol dehydrogenase [Aureimonas sp. Leaf324]** | **10.06** | **5.68E-12** | **9.88E-10** |
| **m.162586** | **dimeric dihydrodiol dehydrogenase, putative [Phytophthora infestans T30-4]** | **8.99** | **3.19E-09** | **3.25E-07** |
| **m.198035** | **Alternative oxidase [Phytophthora nicotianae]** | **8.93** | **1.67E-09** | **1.8E-07** |
| **m.57181** | **bifunctional acetaldehyde-CoA/alcohol dehydrogenase [Thermosynechococcus sp. NK55a]** | **8.81** | **3.25E-09** | **3.3E-07** |
| **m.77775** | **hypothetical protein PHYSODRAFT_358973 [Phytophthora sojae]** | **8.68** | **6.43E-09** | **6.17E-07** |
| **m.254086** | **hypothetical protein L915_21056 [Phytophthora parasitica]** | **8.63** | **1.1E-08** | **9.95E-07** |
| **m.321045** | **6-phosphogluconate dehydrogenase (decarboxylating), partial [Phytophthora parasitica]** | **8.51** | **1.94E-08** | **1.64E-06** |
| **m.181937** | **hypothetical protein PHYSODRAFT_305881 [Phytophthora sojae]** | **8.35** | **2.68E-08** | **2.17E-06** |
| **m.203632** | **peroxiredoxin [Aphanomyces invadans]** | **8.12** | **1.01E-07** | **7.26E-06** |
| **m.294690** | **succinate dehydrogenase, cytochrome b556 subunit [Aphanomyces astaci]** | **7.98** | **1.97E-07** | **0.0000131** |
| **m.39608** | **hypothetical protein PHYSODRAFT_352121 [Phytophthora sojae]** | **7.94** | **2.56E-07** | **0.0000167** |
| **m.138453** | **pyruvate dehydrogenase (acetyl-transferring) E1 component, alpha subunit [Aphanomyces astaci]** | **7.90** | **0.0000002** | **0.0000133** |
| **m.37144** | **unnamed protein product [Albugo laibachii Nc14]** | **7.82** | **4.23E-07** | **0.0000262** |
| **m.16736** | **hypothetical protein PHYSODRAFT_283992 [Phytophthora sojae]** | **7.72** | **9.29E-07** | **0.0000528** |
| **m.37206** | **NADH dehydrogenase flavoprotein 1, mitochondrial precursor [Phytophthora infestans T30-4]** | **7.69** | **9.66E-07** | **0.0000545** |
| **m.99482** | **unnamed protein product [Albugo laibachii Nc14]** | **7.67** | **6.53E-07** | **0.0000383** |
| **m.183628** | **glutathione-disulfide reductase [Aphanomyces astaci]** | **7.64** | **1.39E-06** | **0.0000749** |
| **m.129505** | **hypothetical protein SORBIDRAFT_05g000680 [Sorghum bicolor]** | **7.47** | **1.11E-06** | **0.0000619** |
| **m.106518** | **glyceraldehyde-3-phosphate dehydrogenase, type I, partial [Phytophthora parasitica]** | **7.45** | **2.76E-06** | **0.0001386** |
| **m.121913** | **hypothetical protein PHYSODRAFT_285408 [Phytophthora sojae]** | **7.39** | **0.0000242** | **0.000937** |
| **m.39609** | **hypothetical protein PPTG_09100 [Phytophthora parasitica INRA-310]** | **7.37** | **0.0000078** | **0.0003505** |
| **m.272094** | **succinate dehydrogenase [ubiquinone] iron-sulfur subunit [Saprolegnia diclina VS20]** | **7.19** | **7.58E-06** | **0.0003423** |
| **m.272278** | **manganese superoxide dismutase putative [Albugo laibachii Nc14]** | **7.12** | **0.0000324** | **0.0012105** |
| **m.181668** | **isocitrate dehydrogenase, NADP-dependent [Phytophthora parasitica P1976]** | **6.88** | **0.0000468** | **0.0016592** |
| **m.225391** | **enoyl-ACP reductase [Pedosphaera parvula]** | **6.72** | **0.0001661** | **0.0049238** |
| **m.181849** | **hypothetical protein H310_08590 [Aphanomyces invadans]** | **6.61** | **0.0001954** | **0.0056474** |
| **m.112242** | **hypothetical protein F442_14819 [Phytophthora parasitica P10297]** | **6.61** | **0.0001636** | **0.0048601** |
| **m.273529** | **hypothetical protein BATDEDRAFT_10803 [Batrachochytrium dendrobatidis JAM81]** | **6.58** | **0.0001378** | **0.004218** |
| **m.264186** | **PREDICTED: cytochrome P450 71D8-like [Setaria italica]** | **3.33** | **2.18E-06** | **0.0001116** |
| **m.26238** | **PREDICTED: DIBOA-glucoside dioxygenase BX6-like [Setaria italica]** | **2.91** | **2.67E-14** | **6.33E-12** |
| **m.188036** | **hypothetical protein SORBIDRAFT_05g022340 [Sorghum bicolor]** | **2.87** | **1.31E-13** | **2.85E-11** |
| **m.52678** | **PREDICTED: peroxidase 2-like [Zea mays]** | **2.58** | **4.5E-13** | **9.29E-11** |
| **m.137462** | **hypothetical protein BRADI_4g09040 [Brachypodium distachyon]** | **2.53** | **3.41E-14** | **7.95E-12** |
| **m.105343** | **hypothetical protein SORBIDRAFT_01g007240 [Sorghum bicolor]** | **2.52** | **2.77E-19** | **1.31E-16** |
| **m.244824** | **PREDICTED: thioredoxin M-type, chloroplastic-like [Setaria italica]** | **2.39** | **1.2E-09** | **1.34E-07** |
| **m.237571** | **PREDICTED: peroxidase 2-like [Setaria italica]** | **2.23** | **0.0000136** | **0.0005707** |
| **m.266076** | **hypothetical protein SORBIDRAFT_07g001280 [Sorghum bicolor]** | **2.01** | **1.36E-08** | **0.0000012** |
| **m.150499** | **aldehyde dehydrogenase 5 [Zea mays]** | **1.96** | **7.18E-13** | **1.44E-10** |
| **m.126273** | **PREDICTED: peroxidase 57-like [Setaria italica]** | **1.96** | **0.0003207** | **0.0085632** |
| **m.107171** | **replicase [Lolium latent virus]** | **1.83** | **1.28E-10** | **1.75E-08** |
| **m.64522** | **hypothetical protein SORBIDRAFT_03g034400 [Sorghum bicolor]** | **1.77** | **2.33E-12** | **4.35E-10** |
| **m.245096** | **PREDICTED: peroxidase 1 [Setaria italica]** | **1.76** | **3.19E-12** | **5.82E-10** |
| **m.206325** | **uncharacterized protein LOC107522037 [Zea mays]** | **1.74** | **0.0002303** | **0.0064728** |
| **m.108821** | **PREDICTED: peroxidase 2 [Setaria italica]** | **1.73** | **1.27E-06** | **0.0000693** |
| **m.245166** | **PREDICTED: cytochrome P450 78A9-like [Setaria italica]** | **1.68** | **2.24E-14** | **5.4E-12** |
| **m.14787** | **uncharacterized protein LOC100283169 [Zea mays]** | **1.66** | **1.85E-45** | **7.65E-42** |
| **m.285938** | **PREDICTED: peroxidase 72-like [Setaria italica]** | **1.57** | **4.93E-09** | **4.83E-07** |
| **m.29517** | **PREDICTED: plant cysteine oxidase 2-like [Setaria italica]** | **1.51** | **7.8E-08** | **5.75E-06** |
| **m.151533** | **PREDICTED: cytochrome P450 CYP72A219-like [Setaria italica]** | **1.50** | **0.0001813** | **0.0053016** |
| **m.122847** | **hypothetical protein SORBIDRAFT_10g006050 [Sorghum bicolor]** | **1.47** | **0.0000608** | **0.0020866** |
| **m.29512** | **PREDICTED: plant cysteine oxidase 2-like [Setaria italica]** | **1.45** | **4.64E-15** | **1.2E-12** |
| **m.29518** | **PREDICTED: plant cysteine oxidase 2-like [Setaria italica]** | **1.38** | **8.63E-09** | **8.01E-07** |
| **m.150497** | **hypothetical protein SETIT_000898mg [Setaria italica]** | **1.31** | **0.0002972** | **0.0080241** |
| **m.34834** | **siroheme uroporphyrinogen methyltransferase 1 [Zea mays]** | **1.21** | **6.87E-10** | **8.12E-08** |
| **m.239595** | **hypothetical protein SORBIDRAFT_04g034160 [Sorghum bicolor]** | **1.18** | **1.49E-45** | **6.76E-42** |
| **m.218596** | **PREDICTED: peroxidase 2-like [Setaria italica]** | **1.13** | **1.63E-12** | **3.08E-10** |
| **m.83369** | **PREDICTED: geraniol 8-hydroxylase-like [Setaria italica]** | **1.11** | **4.55E-08** | **3.53E-06** |
| **m.206977** | **uncharacterized protein LOC100273624 [Zea mays]** | **1.05** | **0.0000361** | **0.0013296** |
| **m.203926** | **hypothetical protein SORBIDRAFT_02g034370 [Sorghum bicolor]** | **1.02** | **6.22E-36** | **1.29E-32** |

**Supplemental table 7B. DEGs involved in “oxidation-reduction process” in salt-treated Parish**

| **Gene_ID** | **Description** | **Log_2_FC** | **P value** | **Adjusted P value** |
| --- | --- | --- | --- | --- |
| m.282685 | hypothetical protein SORBIDRAFT_05g003100 [Sorghum bicolor] | 4.38 | 7.78E-19 | 4.77E-17 |
| m.282690 | PREDICTED: cytochrome P450 94C1-like [Setaria italica] | 4.17 | 3.08E-30 | 3.97E-28 |
| m.198771 | putative cytochrome P450 superfamily protein, partial [Zea mays] | 4.01 | 0.0006585 | 0.004989 |
| m.74203 | PsbA (chloroplast) [Bambusa oldhamii] | 3.87 | 0.0000939 | 0.0008965 |
| m.267635 | hypothetical protein SORBIDRAFT_05g003100 [Sorghum bicolor] | 3.84 | 3.61E-15 | 1.56E-13 |
| m.187403 | PREDICTED: 2'-deoxymugineic-acid 2'-dioxygenase-like [Setaria italica] | 3.78 | 1.6E-91 | 2.19E-88 |
| m.283169 | polyphenol oxidase [Setaria italica] | 3.66 | 0.0010859 | 0.0077061 |
| m.204080 | hypothetical protein SORBIDRAFT_01g030560 [Sorghum bicolor] | 3.44 | 6.3E-11 | 1.69E-09 |
| m.154967 | PREDICTED: cytochrome P450 734A6-like [Setaria italica] | 3.30 | 6.18E-23 | 5.02E-21 |
| m.176435 | cytochrome P450 94C1-like [Zea mays] | 3.20 | 1.03E-06 | 0.0000151 |
| m.44266 | hypothetical protein SORBIDRAFT_09g021040 [Sorghum bicolor] | 3.12 | 0.000592 | 0.0045424 |
| m.17513 | PREDICTED: cytochrome P450 734A5-like [Setaria italica] | 3.11 | 5.83E-06 | 0.0000728 |
| m.26238 | PREDICTED: DIBOA-glucoside dioxygenase BX6-like [Setaria italica] | 3.05 | 7.45E-11 | 1.98E-09 |
| m.137462 | hypothetical protein BRADI_4g09040 [Brachypodium distachyon] | 2.93 | 1.36E-08 | 2.64E-07 |
| m.282702 | PREDICTED: cytochrome P450 94C1-like [Setaria italica] | 2.88 | 0.000011 | 0.0001303 |
| m.174331 | NADH dehydrogenase subunit 5 (mitochondrion) [Bambusa oldhamii] | 2.86 | 0.0005402 | 0.0041928 |
| m.88361 | PREDICTED: peroxidase 5-like [Zea mays] | 2.83 | 0.0014216 | 0.0097072 |
| m.59079 | PREDICTED: peroxidase 9-like [Setaria italica] | 2.81 | 0.0003372 | 0.0027726 |
| m.61296 | PREDICTED: putative cytochrome P450 superfamily protein isoform X1 [Zea mays] | 2.81 | 0.0012673 | 0.0087922 |
| m.64522 | hypothetical protein SORBIDRAFT_03g034400 [Sorghum bicolor] | 2.79 | 3.03E-20 | 1.99E-18 |
| m.127911 | PREDICTED: proline dehydrogenase 2, mitochondrial-like [Setaria italica] | 2.74 | 2.89E-145 | 1.72E-141 |
| m.219069 | PREDICTED: L-ascorbate oxidase homolog [Setaria italica] | 2.71 | 0.0000372 | 0.0003882 |
| m.4981 | PREDICTED: peroxidase 25 [Setaria italica] | 2.71 | 1.86E-06 | 0.0000256 |
| m.66489 | PREDICTED: nitrate reductase [NADH] [Setaria italica] | 2.71 | 1.75E-255 | 6.23E-251 |
| m.204071 | hypothetical protein SORBIDRAFT_01g030560 [Sorghum bicolor] | 2.63 | 1.95E-32 | 2.9E-30 |
| m.266076 | hypothetical protein SORBIDRAFT_07g001280 [Sorghum bicolor] | 2.61 | 9.94E-14 | 3.65E-12 |
| m.188036 | hypothetical protein SORBIDRAFT_05g022340 [Sorghum bicolor] | 2.56 | 1.82E-06 | 0.0000251 |
| m.237571 | PREDICTED: peroxidase 2-like [Setaria italica] | 2.55 | 6.86E-06 | 0.0000845 |
| m.113717 | PREDICTED: abscisic acid 8'-hydroxylase 3 [Setaria italica] | 2.55 | 3.54E-30 | 4.53E-28 |
| m.282695 | hypothetical protein SORBIDRAFT_05g003100 [Sorghum bicolor] | 2.50 | 9.65E-15 | 4.03E-13 |
| m.280138 | hypothetical protein SORBIDRAFT_01g001160 [Sorghum bicolor] | 2.48 | 3.07E-14 | 1.2E-12 |
| m.267641 | hypothetical protein SORBIDRAFT_08g003110 [Sorghum bicolor] | 2.48 | 7.06E-28 | 7.79E-26 |
| m.154930 | hypothetical protein SORBIDRAFT_02g040500 [Sorghum bicolor] | 2.47 | 2.13E-38 | 4.25E-36 |
| m.34834 | siroheme uroporphyrinogen methyltransferase 1 [Zea mays] | 2.47 | 1.02E-38 | 2.09E-36 |
| m.160156 | predicted protein [Hordeum vulgare subsp. vulgare] | 2.38 | 5.21E-06 | 0.0000656 |
| m.126273 | PREDICTED: peroxidase 57-like [Setaria italica] | 2.35 | 0.0006227 | 0.0047469 |
| m.245166 | PREDICTED: cytochrome P450 78A9-like [Setaria italica] | 2.33 | 1.63E-20 | 1.09E-18 |
| m.113905 | PREDICTED: probable lipoxygenase 8, chloroplastic [Setaria italica] | 2.30 | 2.32E-13 | 8.16E-12 |
| m.66495 | AChain A, Structural Studies On Corn Nitrate Reductase: Refined Structure Of The Cytochrome B Reductase Fragment At 2.5 Angstroms, Its Adp Complex And An Active Site Mutant And Modeling Of The Cytochrome B Domain | 2.26 | 5.39E-38 | 1.05E-35 |
| m.198183 | PREDICTED: cytochrome P450 72A15-like [Setaria italica] | 2.25 | 3.86E-17 | 2.06E-15 |
| m.239595 | hypothetical protein SORBIDRAFT_04g034160 [Sorghum bicolor] | 2.24 | 2.4E-195 | 4.26E-191 |
| m.52678 | PREDICTED: peroxidase 2-like [Zea mays] | 2.23 | 4.6E-11 | 1.25E-09 |
| m.62488 | Cytochrome P450 99A2 [Aegilops tauschii] | 2.22 | 1.28E-54 | 5.13E-52 |
| m.47963 | unknown [Zea mays] | 2.20 | 4.43E-43 | 1.09E-40 |
| m.127929 | PREDICTED: proline dehydrogenase 2, mitochondrial-like [Setaria italica] | 2.17 | 0.0004364 | 0.0034659 |
| m.50105 | PREDICTED: HIPL1 protein-like [Setaria italica] | 2.15 | 0.0007468 | 0.0055631 |
| m.127936 | hypothetical protein SETIT_035342mg [Setaria italica] | 2.11 | 1.89E-08 | 3.6E-07 |
| m.94094 | hypothetical protein SETIT_029527mg [Setaria italica] | 2.10 | 6.58E-09 | 1.35E-07 |
| m.139442 | hypothetical protein SORBIDRAFT_09g021040 [Sorghum bicolor] | 2.09 | 0.0002488 | 0.0021221 |
| m.26615 | putative cinnamyl-alcohol dehydrogenase family protein [Zea mays] | 2.06 | 1.42E-08 | 2.75E-07 |
| m.129962 | PREDICTED: abscisic acid 8'-hydroxylase 1 [Setaria italica] | 2.03 | 1.94E-75 | 1.87E-72 |
| m.51271 | PREDICTED: uncharacterized protein LOC100381459 isoform X1 [Zea mays] | 2.03 | 0.0001361 | 0.0012449 |
| m.23307 | PREDICTED: cationic peroxidase 1-like [Setaria italica] | 1.97 | 1.26E-21 | 9.17E-20 |
| m.22083 | PREDICTED: cytochrome P450 714C2-like isoform X1 [Brachypodium distachyon] | 1.95 | 1.7E-27 | 1.83E-25 |
| m.173810 | taxane 10-beta-hydroxylase [Zea mays] | 1.94 | 0.0006829 | 0.005154 |
| m.307652 | hypothetical protein SORBIDRAFT_02g023150 [Sorghum bicolor] | 1.94 | 1.65E-06 | 0.0000229 |
| m.151533 | PREDICTED: cytochrome P450 CYP72A219-like [Setaria italica] | 1.91 | 3.27E-06 | 0.0000429 |
| m.285938 | PREDICTED: peroxidase 72-like [Setaria italica] | 1.89 | 2.31E-11 | 6.58E-10 |
| m.51257 | PREDICTED: respiratory burst oxidase homolog protein B-like [Setaria italica] | 1.86 | 0.0000325 | 0.0003442 |
| m.29512 | PREDICTED: plant cysteine oxidase 2-like [Setaria italica] | 1.86 | 6.74E-32 | 9.75E-30 |
| m.8466 | gibberellin 2-beta-dioxygenase [Zea mays] | 1.84 | 1.06E-10 | 2.76E-09 |
| m.150499 | aldehyde dehydrogenase 5 [Zea mays] | 1.78 | 2.74E-09 | 5.93E-08 |
| m.26622 | putative cinnamyl-alcohol dehydrogenase family protein [Zea mays] | 1.78 | 0.000054 | 0.0005445 |
| m.206985 | PREDICTED: tropinone reductase homolog At2g29170-like [Setaria italica] | 1.78 | 0.0005698 | 0.0043875 |
| m.26185 | hypothetical protein SORBIDRAFT_04g030310 [Sorghum bicolor] | 1.78 | 1.85E-12 | 5.9E-11 |
| m.23347 | PREDICTED: peroxidase 4-like [Setaria italica] | 1.77 | 3.94E-48 | 1.22E-45 |
| m.83369 | PREDICTED: geraniol 8-hydroxylase-like [Setaria italica] | 1.77 | 7.78E-27 | 8.02E-25 |
| m.280133 | hypothetical protein SORBIDRAFT_01g001160 [Sorghum bicolor] | 1.76 | 0.000027 | 0.0002903 |
| m.174651 | hypothetical protein SORBIDRAFT_02g040520 [Sorghum bicolor] | 1.73 | 3.22E-13 | 1.11E-11 |
| m.29517 | PREDICTED: plant cysteine oxidase 2-like [Setaria italica] | 1.72 | 8.64E-10 | 2.01E-08 |
| m.198787 | PREDICTED: cytochrome P450 93A2-like [Zea mays] | 1.71 | 2.8E-08 | 5.19E-07 |
| m.226980 | uncharacterized protein LOC100217119 [Zea mays] | 1.70 | 8.78E-108 | 1.95E-104 |
| m.198335 | PREDICTED: 1-aminocyclopropane-1-carboxylate oxidase 1 isoform X1 [Zea mays] | 1.69 | 0.0003365 | 0.0027675 |
| m.173438 | PREDICTED: cytochrome P450 86A1 [Setaria italica] | 1.68 | 5.77E-17 | 3.01E-15 |
| m.187858 | PREDICTED: laccase-10-like [Setaria italica] | 1.66 | 0.000157 | 0.0014156 |
| m.29518 | PREDICTED: plant cysteine oxidase 2-like [Setaria italica] | 1.66 | 5.93E-12 | 1.8E-10 |
| m.94079 | PREDICTED: flavin-containing monooxygenase FMO GS-OX-like 8 [Setaria italica] | 1.59 | 0.0000572 | 0.0005741 |
| m.113684 | PREDICTED: stearoyl-[acyl-carrier-protein] 9-desaturase 1, chloroplastic [Setaria italica] | 1.58 | 1.45E-13 | 5.22E-12 |
| m.248182 | hypothetical protein SORBIDRAFT_04g017460 [Sorghum bicolor] | 1.57 | 1.27E-06 | 0.0000181 |
| m.239478 | hypothetical protein SORBIDRAFT_06g018040 [Sorghum bicolor] | 1.56 | 2.43E-72 | 2.16E-69 |
| m.248363 | PREDICTED: polyphenol oxidase I, chloroplastic-like [Zea mays] | 1.56 | 2.52E-16 | 1.23E-14 |
| m.147368 | PREDICTED: thioredoxin H4-2 [Setaria italica] | 1.55 | 1.54E-12 | 4.96E-11 |
| m.41429 | PREDICTED: flavonol synthase/flavanone 3-hydroxylase [Oryza sativa Japonica Group] | 1.55 | 2.28E-55 | 9.45E-53 |
| m.248170 | hypothetical protein OsI_07154 [Oryza sativa Indica Group] | 1.55 | 1.72E-09 | 3.85E-08 |
| m.56310 | hypothetical protein SORBIDRAFT_01g030560 [Sorghum bicolor] | 1.54 | 1.02E-38 | 2.09E-36 |
| m.206977 | uncharacterized protein LOC100273624 [Zea mays] | 1.54 | 1.03E-06 | 0.0000151 |
| m.41405 | PREDICTED: 1-aminocyclopropane-1-carboxylate oxidase 5-like [Setaria italica] | 1.53 | 1.55E-43 | 3.94E-41 |
| m.226420 | PREDICTED: monothiol glutaredoxin-S2 [Setaria italica] | 1.48 | 0.0010347 | 0.0073781 |
| m.83127 | flavoprotein wrbA [Zea mays] | 1.48 | 4.6E-23 | 3.76E-21 |
| m.98338 | PREDICTED: peroxidase 45-like [Setaria italica] | 1.47 | 1.86E-07 | 3.08E-06 |
| m.307657 | hypothetical protein SORBIDRAFT_02g023150 [Sorghum bicolor] | 1.46 | 0.0007168 | 0.0053697 |
| m.23335 | PREDICTED: peroxidase 4-like [Setaria italica] | 1.46 | 2.8E-46 | 8.15E-44 |
| m.18004 | putative laccase precursor [Zea mays] | 1.45 | 1.1E-17 | 6.12E-16 |
| m.301900 | PREDICTED: L-gulonolactone oxidase-like [Zea mays] | 1.44 | 5.43E-06 | 0.0000682 |
| m.303894 | respiratory burst oxidase protein D variant alpha [Zea mays] | 1.44 | 4.41E-08 | 7.99E-07 |
| m.196099 | hypothetical protein SORBIDRAFT_07g024030 [Sorghum bicolor] | 1.43 | 6.19E-06 | 0.0000769 |
| m.96471 | uncharacterized protein LOC100381459 [Zea mays] | 1.43 | 0.0004642 | 0.0036608 |
| m.185746 | PREDICTED: gibberellin 2-beta-dioxygenase 8-like [Setaria italica] | 1.42 | 2.13E-06 | 0.000029 |
| m.98344 | PREDICTED: peroxidase 45-like [Setaria italica] | 1.42 | 0.000001 | 0.0000146 |
| m.41409 | PREDICTED: 1-aminocyclopropane-1-carboxylate oxidase 5-like [Setaria italica] | 1.40 | 4.14E-62 | 2.68E-59 |
| m.56113 | ascorbate-specific transmembrane electron transporter 1 [Zea mays] | 1.39 | 3.27E-24 | 2.84E-22 |
| m.299765 | PREDICTED: cytochrome P450 CYP72A219-like [Setaria italica] | 1.38 | 2.22E-13 | 7.82E-12 |
| m.158584 | PREDICTED: glutamate dehydrogenase 2-like [Setaria italica] | 1.35 | 4.49E-06 | 0.0000572 |
| m.40826 | catalase [Saccharum hybrid cultivar NCo 376] | 1.34 | 3.14E-13 | 1.09E-11 |
| m.143013 | unknown [Zea mays] | 1.34 | 1.52E-50 | 5.26E-48 |
| m.279952 | PREDICTED: uncharacterized oxidoreductase At1g06690, chloroplastic-like [Setaria italica] | 1.31 | 2.03E-38 | 4.08E-36 |
| m.225221 | PREDICTED: laccase-10-like [Setaria italica] | 1.30 | 0.0000589 | 0.0005889 |
| m.291116 | hypothetical protein SORBIDRAFT_07g024230 [Sorghum bicolor] | 1.29 | 4.2E-09 | 8.85E-08 |
| m.303872 | respiratory burst oxidase protein D variant alpha [Zea mays] | 1.28 | 4.07E-57 | 1.88E-54 |
| m.98341 | hypothetical protein SORBIDRAFT_01g007230 [Sorghum bicolor] | 1.27 | 0.0000163 | 0.0001846 |
| m.265977 | NAD(P)H-dependent oxidoreductase [Zea mays] | 1.27 | 1.01E-11 | 3.01E-10 |
| m.255862 | PREDICTED: peroxidase 16-like [Setaria italica] | 1.26 | 8.32E-10 | 1.94E-08 |
| m.248186 | hypothetical protein SORBIDRAFT_04g017460 [Sorghum bicolor] | 1.26 | 1.68E-09 | 3.76E-08 |
| m.141480 | PREDICTED: glyceraldehyde-3-phosphate dehydrogenase A, chloroplastic [Setaria italica] | 1.26 | 2.64E-22 | 2.04E-20 |
| m.91343 | PREDICTED: probable phospholipid hydroperoxide glutathione peroxidase [Setaria italica] | 1.25 | 0.0001214 | 0.0011261 |
| m.190058 | unknown [Zea mays] | 1.24 | 0.0001114 | 0.0010438 |
| m.136651 | ALDR_HORVURecName: Full=Aldose reductase; Short=AR; AltName: Full=Aldehyde reductase | 1.24 | 0.0001501 | 0.0013606 |
| m.309440 | peroxidase 24 precursor [Zea mays] | 1.23 | 2.45E-25 | 2.3E-23 |
| m.141474 | glyceraldehyde-3-phosphate dehydrogenase A, chloroplastic precursor [Zea mays] | 1.22 | 1.05E-48 | 3.27E-46 |
| m.41396 | PREDICTED: flavanone 3-dioxygenase-like [Setaria italica] | 1.21 | 1.46E-16 | 7.32E-15 |
| m.140358 | PREDICTED: malate dehydrogenase, chloroplastic-like [Setaria italica] | 1.21 | 3.44E-33 | 5.31E-31 |
| m.95209 | PREDICTED: putative respiratory burst oxidase homolog protein H [Setaria italica] | 1.21 | 2.06E-15 | 9.18E-14 |
| m.303885 | PREDICTED: respiratory burst oxidase homolog protein B [Setaria italica] | 1.20 | 3.91E-14 | 1.51E-12 |
| m.95198 | PREDICTED: putative respiratory burst oxidase homolog protein H [Setaria italica] | 1.20 | 5.27E-31 | 7.16E-29 |
| m.154951 | hypothetical protein SORBIDRAFT_02g040490 [Sorghum bicolor] | 1.19 | 0.000688 | 0.0051867 |
| m.2413 | hypothetical protein SORBIDRAFT_03g036760 [Sorghum bicolor] | 1.19 | 1.09E-13 | 3.97E-12 |
| m.139906 | PREDICTED: flavonoid 3'-monooxygenase-like [Setaria italica] | 1.18 | 1.94E-08 | 3.69E-07 |
| m.307287 | hypothetical protein SORBIDRAFT_10g022440 [Sorghum bicolor] | 1.17 | 9.76E-06 | 0.0001165 |
| m.300523 | hypothetical protein SORBIDRAFT_07g022650 [Sorghum bicolor] | 1.17 | 6.47E-06 | 0.00008 |
| m.276373 | hypothetical protein SORBIDRAFT_05g001000 [Sorghum bicolor] | 1.13 | 1.02E-25 | 9.81E-24 |
| m.188251 | PREDICTED: plant cysteine oxidase 5-like [Setaria italica] | 1.12 | 1.96E-15 | 8.79E-14 |
| m.199448 | hypothetical protein SORBIDRAFT_02g040190 [Sorghum bicolor] | 1.12 | 5.61E-11 | 1.51E-09 |
| m.72825 | PREDICTED: flavonol synthase/flavanone 3-hydroxylase-like [Zea mays] | 1.12 | 1.61E-30 | 2.13E-28 |
| m.299275 | uncharacterized protein LOC100281213 [Zea mays] | 1.11 | 7.97E-24 | 6.81E-22 |
| m.187156 | hypothetical protein SORBI_001G062300 [Sorghum bicolor] | 1.11 | 4.84E-30 | 6.17E-28 |
| m.41447 | PREDICTED: flavanone 3-dioxygenase-like [Setaria italica] | 1.09 | 6.07E-17 | 3.15E-15 |
| m.203926 | hypothetical protein SORBIDRAFT_02g034370 [Sorghum bicolor] | 1.09 | 8.06E-42 | 1.87E-39 |
| m.14298 | PREDICTED: extradiol ring-cleavage dioxygenase-like [Zea mays] | 1.08 | 1.28E-20 | 8.67E-19 |
| m.27825 | hypothetical protein SETIT_019843mg, partial [Setaria italica] | 1.08 | 0.0003571 | 0.0029166 |
| m.150415 | PREDICTED: peroxidase 11 [Setaria italica] | 1.08 | 1.15E-21 | 8.45E-20 |
| m.5429 | acc oxidase [Zea mays] | 1.06 | 0.0000828 | 0.0008019 |
| m.124640 | hypothetical protein SORBIDRAFT_06g032450 [Sorghum bicolor] | 1.06 | 5.95E-69 | 4.81E-66 |
| m.216101 | hypothetical protein SORBIDRAFT_02g036650 [Sorghum bicolor] | 1.06 | 3.93E-06 | 0.0000507 |
| m.266697 | hypothetical protein SORBIDRAFT_10g006650 [Sorghum bicolor] | 1.03 | 2.58E-13 | 9.05E-12 |
| m.145682 | PREDICTED: peroxidase 21 [Setaria italica] | 1.03 | 0.0000589 | 0.0005895 |
| m.18076 | gibberellin 2-beta-dioxygenase [Saccharum hybrid cultivar R570] | 1.02 | 0.0000265 | 0.0002856 |
| m.232330 | PREDICTED: fatty acid desaturase DES2 [Setaria italica] | 1.02 | 7.88E-27 | 8.1E-25 |
| m.83132 | flavoprotein wrbA [Zea mays] | 1.02 | 9.21E-06 | 0.0001105 |
| m.41387 | PREDICTED: flavanone 3-dioxygenase-like [Setaria italica] | 1.01 | 7.1E-16 | 3.3E-14 |
| m.226979 | uncharacterized protein LOC100217119 [Zea mays] | 1.01 | 1.62E-08 | 3.11E-07 |

**Supplemental table 8A. DEGs with “nucleic acid binding activity” in salt-treated Supreme**

| **Gene_ID** | **Description** | **Log_2_FC** | **P value** | **Adjusted P value** |
| --- | --- | --- | --- | --- |
| m.268973 | splicing factor putative [Albugo laibachii Nc14] | 10.39 | 9.13E-13 | 1.8E-10 |
| m.95962 | hypothetical protein L917_04771 [Phytophthora parasitica] | 7.20 | 0.000178281 | 0.005223068 |
| m.326868 | hypothetical protein L915_18980 [Phytophthora parasitica] | 6.97 | 0.000044 | 0.001570743 |
| m.319487 | DEAD-box ATP-dependent RNA helicase 56 [Aphanomyces invadans] * | 6.92 | 0.0000824 | 0.002708217 |
| m.71991 | hypothetical protein SETIT_017165mg [Setaria italica] | 5.72 | 0.000218737 | 0.006232131 |

**Supplemental table 8B. DEGs with “nucleic acid binding activity” in salt-treated Parish**

| **Gene_ID** | **Description** | **Log_2_FC** | **P value** | **Adjusted P value** |
| --- | --- | --- | --- | --- |
| m.73458 | PREDICTED: endonuclease 2-like [Setaria italica] | 1.26 | 0.000978368 | 0.007035904 |
| m.226928 | TPA: hypothetical protein ZEAMMB73_851898 [Zea mays] | 1.66 | 5.60E-07 | 8.62E-06 |
| m.292931 | PREDICTED: uncharacterized protein LOC101753419 [Setaria italica] | 1.72 | 1.42E-09 | 3.20E-08 |
| m.159032 | AF466646_7putative polyprotein [Zea mays] | 1.77 | 1.42E-08 | 2.76E-07 |
| m.292921 | PREDICTED: uncharacterized protein LOC101753419 [Setaria italica] | 1.78 | 9.77E-67 | 7.24E-64 |
